# Predicting viral exposure response from modeling the changes of co-expression networks using time series gene expression data

**DOI:** 10.1186/s12859-020-03705-0

**Published:** 2020-08-26

**Authors:** Fangli Dong, Yong He, Tao Wang, Dong Han, Hui Lu, Hongyu Zhao

**Affiliations:** 1 School of Mathematical Sciences, Shanghai Jiao Tong University, Dongchuan Road, Shanghai, 200240 China; 2 SJTU-Yale Joint Center of Biostatistics and Data Science, Shanghai Jiao Tong University, Dongchuan Road, Shanghai, 200240 China; 3 Institute for Financial Studies, Shandong University, No. 27 Shanda South Road, Jinan, 250100 China; 4 School of Life Sciences and Biotechnology, Shanghai Jiao Tong University, Dongchuan Road, Shanghai, 200240 China; 5 Department of Biostatistics, Yale School of Public Health, 60 College Street, New Haven, CT 06520 USA

**Keywords:** Change point, Kernel method, Time-series gene expression data, Co-expression networks, Dynamic information, Model interpretation

## Abstract

**Background:**

Deciphering the relationship between clinical responses and gene expression profiles may shed light on the mechanisms underlying diseases. Most existing literature has focused on exploring such relationship from cross-sectional gene expression data. It is likely that the dynamic nature of time-series gene expression data is more informative in predicting clinical response and revealing the physiological process of disease development. However, it remains challenging to extract useful dynamic information from time-series gene expression data.

**Results:**

We propose a statistical framework built on considering co-expression network changes across time from time series gene expression data. It first detects change point for co-expression networks and then employs a Bayesian multiple kernel learning method to predict exposure response. There are two main novelties in our method: the use of change point detection to characterize the co-expression network dynamics, and the use of kernel function to measure the similarity between subjects. Our algorithm allows exposure response prediction using dynamic network information across a collection of informative gene sets. Through parameter estimations, our model has clear biological interpretations. The performance of our method on the simulated data under different scenarios demonstrates that the proposed algorithm has better explanatory power and classification accuracy than commonly used machine learning algorithms. The application of our method to time series gene expression profiles measured in peripheral blood from a group of subjects with respiratory viral exposure shows that our method can predict exposure response at early stage (within 24 h) and the informative gene sets are enriched for pathways related to respiratory and influenza virus infection.

**Conclusions:**

The biological hypothesis in this paper is that the dynamic changes of the biological system are related to the clinical response. Our results suggest that when the relationship between the clinical response and a single gene or a gene set is not significant, we may benefit from studying the relationships among genes in gene sets that may lead to novel biological insights.

## Background

In genomics studies, time-series gene expression data [1–3] often need to be processed and analyzed. In 2016, DREAM CHALLENGES released an open challenge called ‘Respiratory Viral DREAM Challenge: Discovering dynamic molecular signatures in response to virus exposure’ (https://www.synapse.org/#!Synapse:syn5647810/wiki/399108). The aim was to develop early predictors of susceptibility and contagiousness based on expression profiles collected prior to and at early time points following viral exposure. Some work reported the differences of transcriptomics [4–6] in the host response between symptomatic and asymptomatic subjects exposure to respiratory viruses. Additionally, as what were done by most participants (https://www.synapse.org/#!Synapse:syn5647810/wiki/402364), some common machine learning algorithms [7] can be used if we treat the challenge as a prediction problem. The challenge results [see Additional file 1 for parts of the challenge results] demonstrate that the prediction performance significantly depends on the participants’ models. However, we need to average the time series data across time or only use cross-sectional data at a time to perform ensemble learning, and the dynamic information of the time series data is lost in these approaches. Moreover, in the early stage of infection (within 24 h), there is little separation of the trajectories of genes among subjects with different clinical responses. Previous studies [8, 9] also showed that the individual responses after exposure to respiratory virus are influenced not only by the baseline immune status of the host but also by the dynamics of the early host immune response immediately following exposure. If we only consider a single gene, there is no distinct pattern in both cross-sectional and dynamic data. It is difficult to differentiate between positive and negative groups by gene expression levels at early stage. In this paper, we resort to gene sets analysis to correlate exposure response with dynamic gene expression patterns in gene sets. To consider multiple genes, some methods have been proposed to infer the relationship between genes. For example, the Dynamic Bayesian Network (DBN) was used to establish the dynamic regulatory network [10]. We note that a number of groups have studied time-varying dynamic Bayesian networks (TV-DBN) to model the varying network structures and reveal the dynamics of biological systems [11, 12]. The dynamic mixed membership stochastic block model (dMMSB) helps to infer the biological functions of genes through modeling the dynamic tomography of networks [13]. The review of differential network biology [14] advocated that differential network mapping at large scales may provide a deeper understanding of complex biological phenomena. The work [15] analyzed multiple differential co-expression networks based on time-course RNA-Seq data. Through Multiple Differential Modules (M-DMs), they found that dynamic modules are associated with the development of heart failure. These results in the literature suggest that considering the dynamics of networks may help us to better understand disease onset and progression. However, how to extract useful dynamic information from time-series gene expression data to build predictive model remains a challenging problem.

To study the relationship between viral exposure response and time-series gene expression data, we hypothesize that the changes (i.e. dynamics) of the relationship between genes in gene sets may be informative about viral exposure response, and propose a statistical framework to characterize and integrate dynamic information for response prediction where the model parameters have clear biological interpretations. The main innovations of the paper are: Firstly, we use spectral norm to extract information of the difference between two networks. Secondly, we model the changes of dynamic co-expression networks based on the graph-based change point detection method. Thirdly, we measure the similarity between two subjects by the relationship between gene trajectories.

The rest of the paper is organized as follows: In the “Results” Section, we evaluate the performance of our method using both simulated and real data. The results include data description and preprocessing, preliminary analysis and inference results. This is followed by the “Discussion” and “Conclusions” Sections. The “Methods” Section first introduces the notations, then describes the statistical models and inference procedure proposed in this manuscript.

## Results

### Simulations

In this section we assess the performance of the proposed algorithm on the data simulated as follows. For simplicity, we fixed the number of genes *G*=80. The sample size *N* and total time points *T* took values from the sets {20,50,100} and {40,80,150}, respectively. In the main text, we show the evaluation results under the case {*N*=100,*T*=40}. For the other cases, the results are provided in the Supplementary Materials [see Additional file 1]. We partition these 80 genes into four gene sets indexed by *O*_1_, *O*_2_, *O*_3_ and *O*_4_, respectively, with each gene set containing 20 genes. To model the time series data, we assume an *AR*(1) model for the mean expression levels, i.e.

$$\boldsymbol{\mu}_{t}=0.5\boldsymbol{\mu}_{t-1}+\boldsymbol{\varepsilon}_{t},~~\boldsymbol{\mu}_{0}=\boldsymbol{0},~~\boldsymbol{\varepsilon}_{t}\sim \mathcal{N}\left(\boldsymbol{0},\Sigma_{0.1}\right),~~t=1,...,T, $$

where *Σ*_0.1_ is the diagonal matrix with 0.1 as the diagonal element. In our model, the algorithm is based on the relationship between the response label and the change of the dynamic structure. As described in the “Models” section, we assume that under the null hypothesis, the covariance matrix of the simulated data is *Σ*_0_ across the time points and under the alternative hypothesis, the covariance matrix is *Σ*_0_ up to some time point after which it changes to *Σ*_1_. We assume $\Sigma =\mathbbm {I}+ \rho \cdot \mathbbm {1}- diag(\rho)$ where *ρ* is a constant and ***1*** is the matrix of 1. For the null hypothesis, *ρ*=0 and we consider different scenarios for the alternative hypothesis when *ρ* takes value from set {0.1,0.3,0.5,0.7,0.9}. The time-series gene expression data are simulated for 50 subjects labelled ‘ +1’ through the model,

- $\boldsymbol {x}_{iO_{1}t_{1}} \sim \mathcal {N}\left (\boldsymbol {\mu }_{t}, \Sigma _{0}\right),~~i=1,...,50,~t_{1}=1,...,15$,
  $\boldsymbol {x}_{iO_{1}t_{2}} \sim \mathcal {N}\left (\boldsymbol {\mu }_{t}, \Sigma _{1}\right),~~i=1,...,50,~t_{2}=16,...,40$,
- $\boldsymbol {x}_{iO_{2}t} \sim \mathcal {N}\left (\boldsymbol {\mu }_{t}, \Sigma _{0}\right),~~i=1,...,50,~t=1,...,40$,
- $\boldsymbol {x}_{iO_{3}t_{1}} \sim \mathcal {N}\left (\boldsymbol {\mu }_{t}, \Sigma _{1}\right),~~i=1,...,50,~t_{1}=1,...,25$,
  $\boldsymbol {x}_{iO_{3}t_{2}} \sim \mathcal {N}\left (\boldsymbol {\mu }_{t}, \Sigma _{0}\right),~~i=1,...,50,~t_{2}=26,...,40$,
- $\boldsymbol {x}_{iO_{4}t} \sim \mathcal {N}\left (\boldsymbol {\mu }_{t}, \Sigma _{1}\right),~~i=1,...,50,~t=1,...,40$.

For 50 subjects labelled ‘ −1’, the data are generated by

- $\boldsymbol {y}_{jO_{1}t_{1}} \sim \mathcal {N}\left (\boldsymbol {\mu }_{t}, \Sigma _{0}\right),~~j=51,...,100,~t_{1}=1,...,15$,
  $\boldsymbol {y}_{jO_{1}t_{2}} \sim \mathcal {N}\left (\boldsymbol {\mu }_{t}, \Sigma _{1}\right),~~j=51,...,100,~t_{2}=16,...,40$,
- $\boldsymbol {y}_{jO_{2}t_{1}} \sim \mathcal {N}\left (\boldsymbol {\mu }_{t}, \Sigma _{0}\right),~~j=51,...,100,~t_{1}=1,...,20$,
  $\boldsymbol {y}_{jO_{2}t_{2}} \sim \mathcal {N}\left (\boldsymbol {\mu }_{t}, \Sigma _{1}\right),~~j=51,...,100,~t_{2}=21,...,40$,
- $\boldsymbol {y}_{jO_{3}t_{1}} \sim \mathcal {N}\left (\boldsymbol {\mu }_{t}, \Sigma _{1}\right),~~j=51,...,100,~t_{1}=1,...,25$,
  $\boldsymbol {y}_{jO_{3}t_{2}} \sim \mathcal {N}\left (\boldsymbol {\mu }_{t}, \Sigma _{0}\right),~~j=51,...,100,~t_{2}=26,...,40$,
- $\boldsymbol {y}_{jO_{4}t_{1}} \sim \mathcal {N}\left (\boldsymbol {\mu }_{t}, \Sigma _{1}\right),~~j=51,...,100,~t_{1}=1,...,20$,
  $\boldsymbol {y}_{jO_{4}t_{2}} \sim \mathcal {N}\left (\boldsymbol {\mu }_{t}, \Sigma _{0}\right),~~j=51,...,100,~t_{2}=21,...,40$,

where $\boldsymbol {x}_{iO_{p}t}=\left \{x_{igt}: g\in O_{p}\right \}$, $\boldsymbol {y}_{iO_{p}t}=\left \{y_{igt}:g\in O_{p}\right \}$ and *p*∈{1,2,3,4}. Under our simulation models, we know that the first and third gene sets have changes in both the positive and negative groups, and the changes happen at time points 15 and 25, respectively. For the second and fourth gene sets, the positive group has no change point and the negative group has changes at the 20th time point. Therefore, the second and fourth gene sets are informative about the response label. We compared the proposed algorithm with commonly used machine learning algorithms, including Logistic Regression (LR), Linear Discriminant Analysis (LDA), Support Vector Machine (SVM) and K-Nearest Neighbor (KNN) [7]. Note that LR is with lasso penalty. The prior probabilities of class membership in LDA use the class proportions in the training set. The kernel function in SVM is the radial basis function, $\exp \left (-\frac {|u|^{2}}{G}\right)$. The number of neighbors in KNN is set to 3. We perform 100 simulations, and for each simulation we randomly select 70% subjects as the training set, and the remaining as the test set. We evaluate the performance of the proposed algorithm from three aspects: change point detection, parameter inference, and prediction accuracy, respectively.

**Change point detection and parameter inference** The results under different scenarios are shown in Table 1. We have 4 gene sets indexed 1, 2, 3, and 4, respectively and the inferred parameters of these 4 gene sets are *b*_1_, *b*_2_, *b*_3_, and *b*_4_, respectively. As discussed in the simulation models, the subject label is the result of different change points in gene sets 2 and 4. For comparability, the absolute value of parameter ***b*** is denoted by |***b***|. When *ρ* is greater than 0.3, |*b*_2_| and |*b*_4_| are the largest in the 4 parameters which is consistent with the model structure. So when the difference between *Σ*_0_ and *Σ*_1_ is large enough, our method can identify the gene sets which contribute more to the response label. In Table 1, ‘CHP’ represents the average value over 100 replications for the estimation of change-point position, with the standard deviation in the parentheses. ‘*P*-value’ is the average *p*-value over 100 replications using graph-based change point detection method. ‘CHP(%)’ represents the proportion of times the change point is precisely detected in 100 simulations. When *ρ* is less than 0.1, the structure difference between *Σ*_0_ and *Σ*_1_ is small, and the detected change point may not be statistically significant. When *ρ* is greater than 0.5, there is more than 90% chance to detect the change point.

**Table 1:** Results under different scenarios

	*ρ*=0.1				*ρ*=0.3				*ρ*=0.5	
Set index	1	2	3	4	1	2	3	4	1	2
|***b***|	0.13(0.05)	0.26(0.06)	0.13(0.05)	0.04(0.06)	0.07(0.02)	0.23(0.05)	0.07(0.02)	0.11(0.05)	0.06(0.01)	0.20(0.03)
CHP ^*†*^	15.21(0.62)	20.85(12.43)	24.58(3.70)	19.84(8.89)	15.05(0.22)	20.00(0.00)	24.97(0.17)	19.68(4.02)	15.06(0.28)	20.00(0.00)
*P*-value ^*‡*^	0.00	0.09	0.00	7.13 ×10^−3^	0.00	0.00	0.00	9.59 ×10^−4^	0.00	0.00
CHP(%) ^*§*^	0.88	0.11	0.73	0.47	0.95	1.00	0.97	0.73	0.95	1.00
AUC	0.58(0.06)				0.60(0.06)				0.65(0.08)	
	*ρ*=0.5		*ρ*=0.7				*ρ*=0.9			
Set index	3	4	1	2	3	4	1	2	3	4
|***b***|	0.05(0.01)	0.11(0.05)	0.04(0.01)	0.19(0.02)	0.04(0.01)	0.12(0.04)	0.04(0.01)	0.17(0.01)	0.03(0.01)	0.12(0.03)
CHP	24.91(0.29)	19.85(1.77)	15.03(0.17)	20.00(0.00)	24.95(0.22)	19.67(2.36)	15.03(0.17)	20.00(0.00)	24.97(0.17)	20.30(0.32)
*P*-value	0.00	8.78 ×10^−4^	0.00	0.00	0.00	0.00	0.00	0.00	0.00	6.32 ×10^−4^
CHP(%)	0.91	0.88	0.97	1.00	0.95	0.90	0.97	1.00	0.97	0.92
AUC			0.71(0.08)				0.90(0.07)			

The standard deviations are in the parentheses.

^*†*^CHP (Change Point Position) is the average value over 100 replications for the estimation of change-point position.

^*‡*^When *P*-value is less than 10^−10^, we set it to 0.00.

^*§*^CHP(%) represents the proportion of times the change point is precisely detected over 100 simulations.

**Fig. 1:**
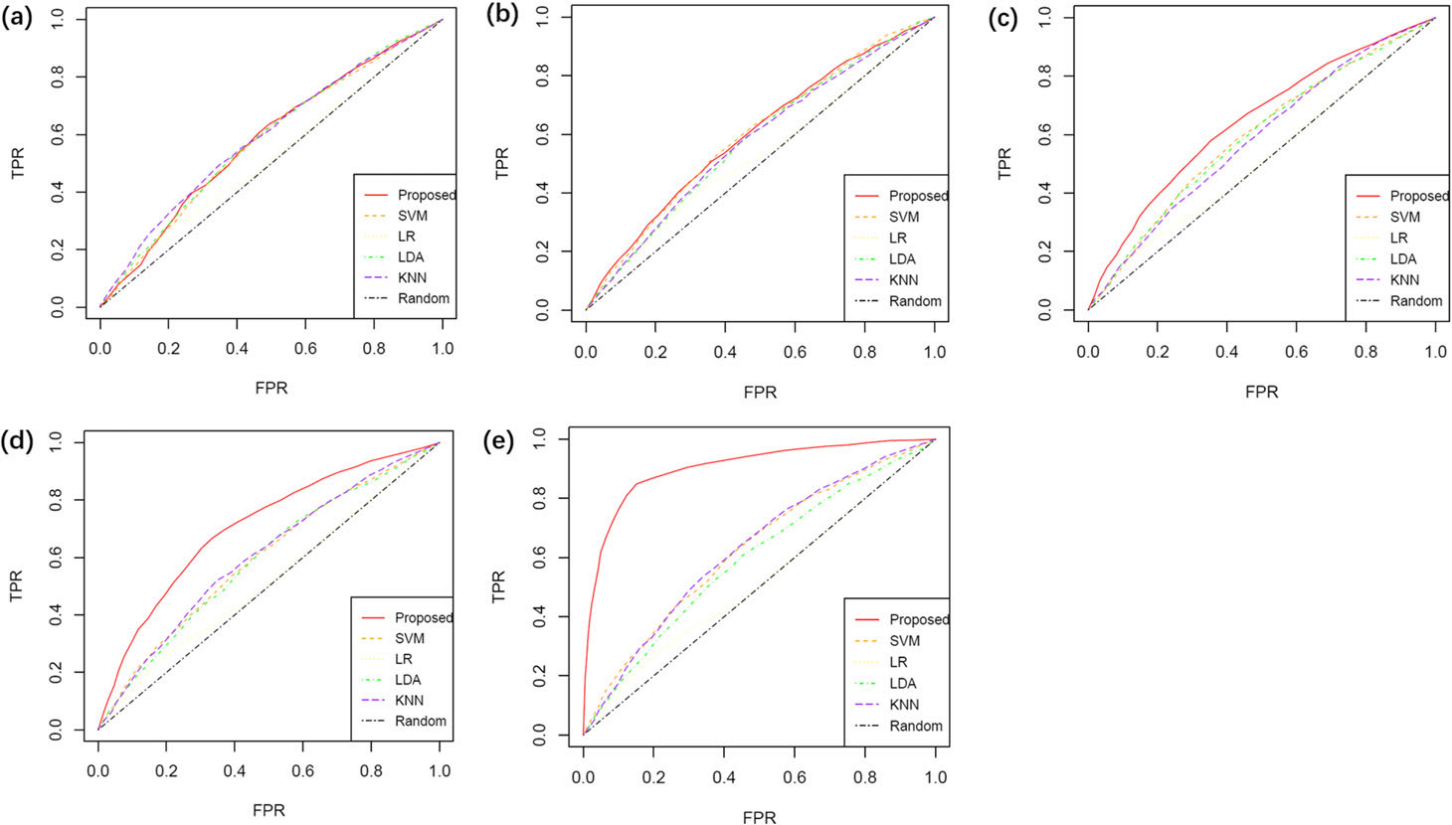
ROC curves. When *ρ* takes different values from {0.1,0.3,0.5,0.7,0.9}, average ROC curves over 100 replications of different algorithms are shown. **a** *ρ*=0.1. When the difference between *Σ*_0_ and *Σ*_1_ is small, the positive and negative groups are difficult to distinguish. The performances of all algorithms are similar. **b** *ρ*=0.3. The results are similar to that of *ρ*=0.1. **c** *ρ*=0.5. Our algorithm is slightly better than the others. **d** *ρ*=0.7. The proposed algorithm outperforms the other algorithms. **e** *ρ*=0.9. The performance of the proposed algorithm is substantially better than the others

**Prediction accuracy** We average the time series data across time points as the input before they are analyzed by LR, LDA, SVM and KNN. The average ROC curves over 100 simulations of the classification results for each algorithm are shown in Fig. 1, where ‘FPR’ represents false positive rate and ‘TPR’ represents true positive rate. We can see that there is more advantage of our method with an increasing value of *ρ*. The average AUC values are summarized in Table 2. The proposed algorithm has the highest average AUC value of 100 simulations when *ρ* is greater than 0.5. Moreover, the ‘AUC’ row of Table 1 shows the classification performance for the test set. We can see that the value of AUC increases with the increase of *ρ*, which is consistent with our model hypothesis, as it is easier to infer the labels with a larger *ρ*.

**Table 2:** AUC of different algorithms under different scenarios

	*ρ*	Proposed	SVM	LR	LDA	KNN
	0.1	0.58(0.06)	0.58(0.06)	0.55(0.07)	0.59(0.07)	0.59(0.06)
	0.3	0.60(0.06)	0.60(0.06)	0.55(0.07)	0.58(0.07)	0.58(0.06)
	0.5	0.65(0.08)	0.60(0.07)	0.56(0.06)	0.59(0.07)	0.59(0.07)
	0.7	0.71(0.08)	0.60(0.07)	0.56(0.06)	0.59(0.07)	0.60(0.07)
	0.9	0.90(0.07)	0.63(0.08)	0.56(0.06)	0.60(0.06)	0.63(0.09)

Standard deviations are in the parenthesis.

As described in the “Methods” section, our algorithm requires the given gene sets as input. So the performance of our algorithm may be affected by the way of grouping genes. We evaluated the performance of our algorithm in different ways of grouping genes. The AUC and prediction accuracy may depend on the grouping method, where a higher enrichment of signals in the pre-defined gene sets will lead to better performance as expected. More details are provided in the Supplementary Materials [see Additional file 1].

### Real data analysis

#### Data description and preprocessing

**Fig. 2:**
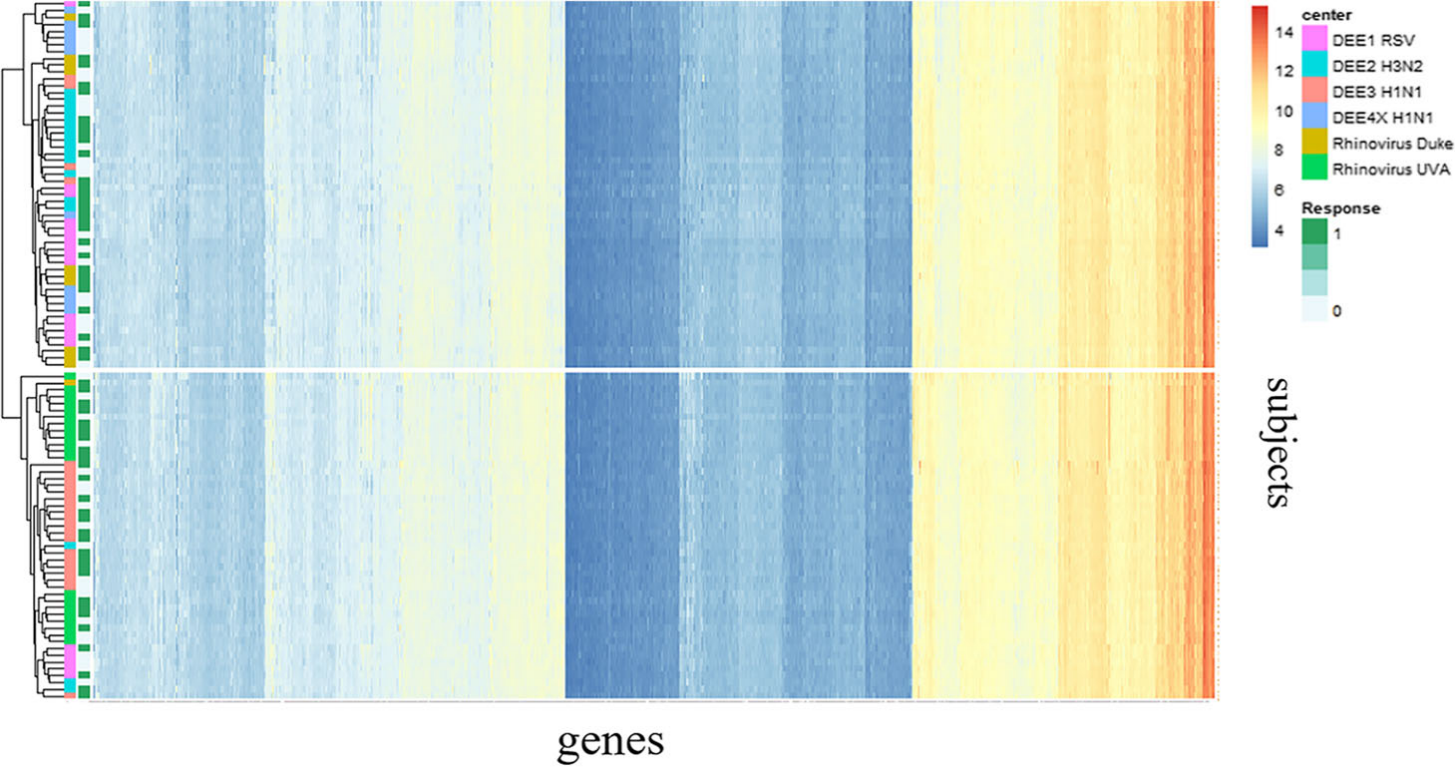
Heat map of the baseline gene expression data across subjects. Genes are clearly classified into different groups. The data seem to suggest two groups with subtle differences, but different viral response groups are not clearly separated with these two groups. On the other hand, centers DEE3 H1N1 and Rhinovirus UVA are in the same group, whereas the remaining centers are in the other group suggesting center/batch effects

In this section, we evaluate the performance of our proposed method through real data analysis. Some challenge results related to this paper are provided in the Supplementary Materials [see Additional file 1]. The complete results can be found at the URL (https://www.synapse.org/#!Synapse:syn5647810/wiki/402364) (note that only the registered users can log into the website). The time-series gene expression data for this challenge were collected from healthy volunteers exposed to a respiratory virus within a controlled experimental setting where some became ill and others did not despite the same exposure. Data were derived from seven viral challenge experiments in which volunteers were exposed to one of four different respiratory viruses (Influenza H1N1, Influenza H3N2, Respiratory Syncytial Virus, or Rhinovirus) in order to find gene expression profiling signatures of susceptibility. Peripheral blood gene expression profiling was made at 55 time points ranging between -30 h (pre-exposure) and 672 h (post-exposure). The released data include 125 subjects from seven study centers with time-series gene expression data for 22,277 probes in peripheral blood for each subject, with a total of 2371 samples. Additionally, clinical information was also available, such as age, gender, and the time of samples measured. To reduce noise, we removed 7 subjects who were injected interfering viruses, and removed probes corresponding to multiple genes, and averaged the multiple probes corresponding to the same gene. We considered a total of 12,532 genes. Therefore, we have *N*=118, *G*=12,532, and *T*=55 for this data set. There are 68 subjects with positive labels and 50 subjects with negative labels. The overall data can be visualized by the heat map as shown in Fig. 2. We can see that the genes can be grouped into distinct clusters, while samples can not be clustered according to response. Moreover, different study centers are clustered together, suggesting possible batch effects. In the following analysis, we normalized the gene expression data according to each time point to remove batch effects.

#### Preliminary analysis

**Fig. 3:**
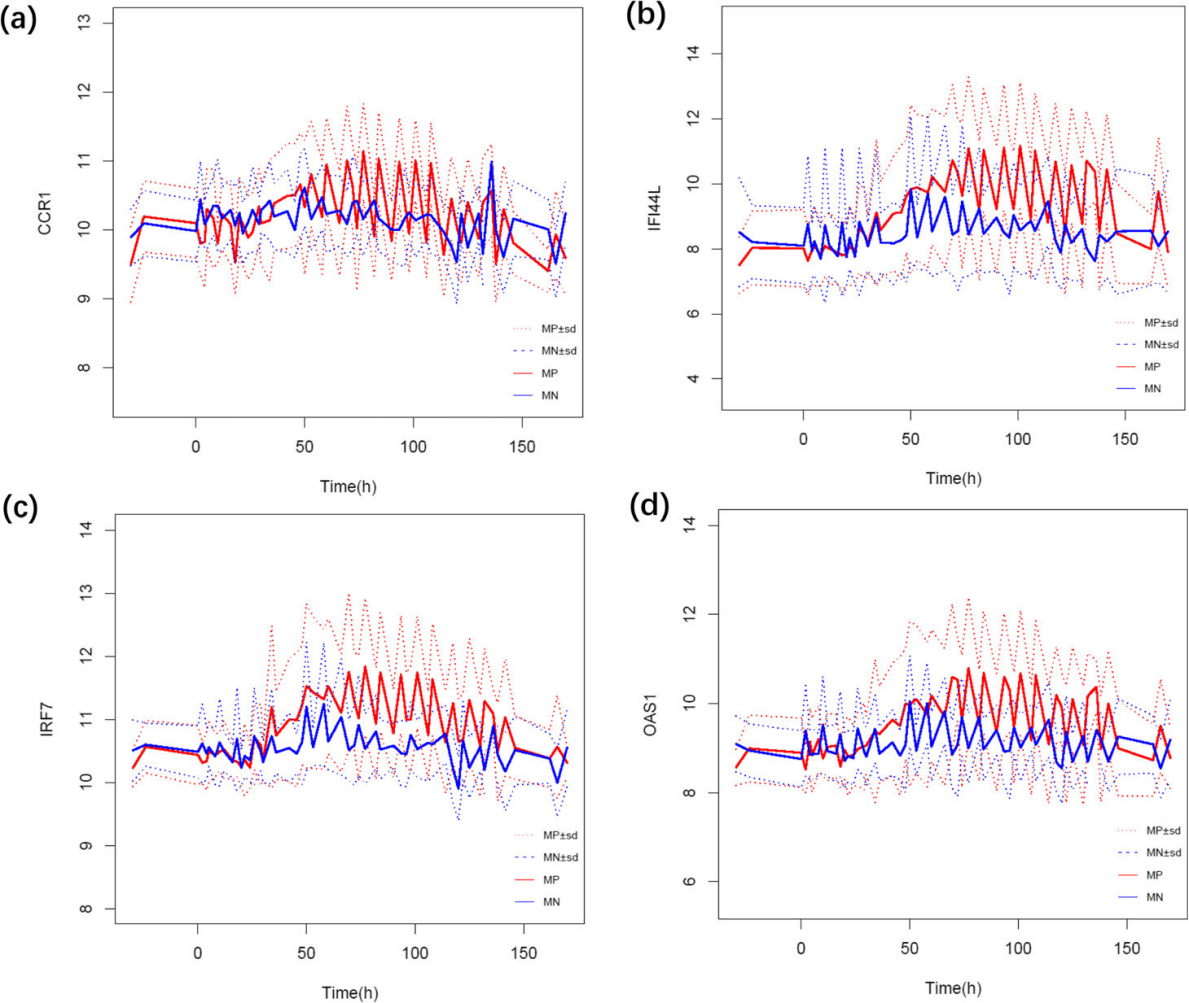
Time series plots of four distinct genes. “MP” and “NP” represent the means of the positive and negative groups, respectively. “sd” means the standard deviations. **a** For gene CCR1, the positive and negative groups are difficult to distinguish. On average, the negative group is below the positive group in the middle stage. **b** For gene IFI44L, the positive group has an obvious upward pattern at about the 50th hour. However it is difficult to distinguish the positive and negative groups in early stage (within 24 h). **c** For gene IRF7, the pattern is similar to that of gene IFI44L. **d** For gene OAS1, at the 50th hour or so, the positive and negative groups can be better distinguished. However there is little separation of the trajectories of these genes for the positive and negative groups in the early stage

A number of studies [16, 17] reported the differences in the host response between symptomatic and asymptomatic subjects challenged with respiratory viruses. For simplicity, we call the symptomatic response group the positive group and the asymptomatic response group the negative group. Firstly, we analyzed the cross-sectional data and performed differential expression analysis at a single time point. No significant difference in single gene expression level was found between the positive and negative groups before 40 h. We further investigated the relationship between gene trajectories and responses. Some papers [9, 17, 18] reported that OAS1, IFI44L, IRF7 and CCR1 may be associated with the response. From the expression trajectories of OAS1, IFI44L, IRF7 and CCR1 shown in Fig. 3, we can see that the changes of expression variances for OAS1, IFI44L, IRF7 occur at latter stage and expression variance of CCR1 does not change over time. That is as a single dynamic time series for these related genes, the positive and negative groups have significant differences after 45 h, however they do not exhibit differences at the early stage (within 24 h). Therefore, if we only consider a single gene, there is no distinct pattern in both cross-sectional and dynamic data. Therefore, it is difficult to differentiate between positive and negative groups by gene expression levels at early stage. On the other hand, the paper [19] shows that viral shedding increases sharply between 0.5 and 1 day (within 24 h) after exposure and consistently peaks on day 2. We resort to gene sets analysis to correlate exposure response with dynamic gene expression patterns in gene sets.

Firstly, we selected the gene sets that may be related to viral exposure responses. We consider “SYMPTOMATIC-SC2” as the response label which is a binary variable indicating post-exposure maximum symptom score greater than six and then screen out differentially expressed genes from each cross-sectional gene expression data at 55 time points, even if it is not significant. This led to 55 gene sets. Secondly, for each gene set, we represent it as an undirected weighted network and the weight is given by gene expression similarity, where we used the Pearson correlation coefficient of two genes to define their similarity. That is the function *h* in the “Models” section is Pearson correlation coefficient. We have tried a number of definitions of similarity and Pearson correlation had better performance overall. For each gene set, we obtained 55 time-dependent networks. And we detected change points for these networks using the method introduced in the “Models” section. After change point detection, all gene sets are sorted according to the time of change point. Thirdly, we set up multiple kernel prediction model based on the gene sets in which the relationships among genes change at early stage. Each gene set is integrated into a kernel.

#### Results

**Fig. 4:**
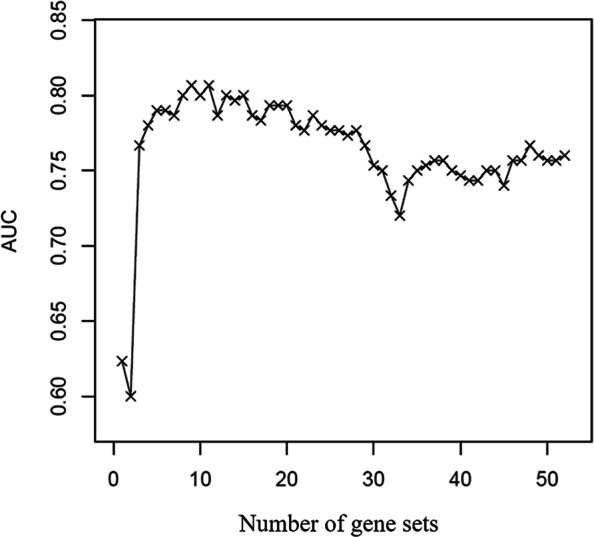
AUC. The value of AUC as a function of the number of genes considered. Gene sets are added to the Bayesian multiple kernel model one by one according to the order of the time when the change point occurs. It can be seen that the AUC increases at the beginning, and has the highest value when the 12th gene set is included. It then oscillates downward as more gene sets are added until the 35th gene set, when AUC starts to increase again

We randomly selected 70% subjects as the training set and the remaining as the test set. The training set contained 83 subjects (35 subjects with negative label and 48 subjects with positive label), 12,532 genes, and up to 55 time points. We want to test the biological hypothesis that the dynamic networks with early change point contribute more to the response label. Figure 4 shows the prediction performance of the model for the test set when we added the gene sets in the order of the detected change point time. It can be seen from Fig. 4 that at the early stage, with more gene sets included, AUC increased. However, after more than 12 gene sets were included, AUC started to decrease, which indicates that an increasing number of gene sets does not lead to an increase of prediction accuracy. This is consistent with our hypothesis that networks that change in the early stages are associated with the response label. Moreover, the curve in Fig. 4 has a turning point at the 35th gene set when AUC starts to increase again, suggesting that those unchanged gene sets may also have information on exposure response. This may be because those unchanged gene sets are markers of the asymptomatic group, which is consistent with the stable performance of the negative group in Fig. 3. Next, we investigated the learning parameter vector ***b***. In terms of 55 gene sets, we consider those gene sets among the top 12 in which the relationships among genes change at early stage. The inferred parameters are summarized in Table 3.

**Table 3:** Learning parameters for gene sets ^*$*^

	Gene set index ^*¶*^	44	2	34	35	25	22
	|***b***|	0.101	0.101	0.101	0.098	0.096	0.095
	Gene set index	18	43	8	30	37	20
	|***b***|	0.094	0.088	0.086	0.085	0.084	0.080

^*$*^In 55 gene sets, these 12 gene sets are among the top 12 in terms of the change point time.

^*¶*^At each time point, through differential expression analysis, we obtain a gene set, and we use time point to index the gene set.

**Fig. 5:**
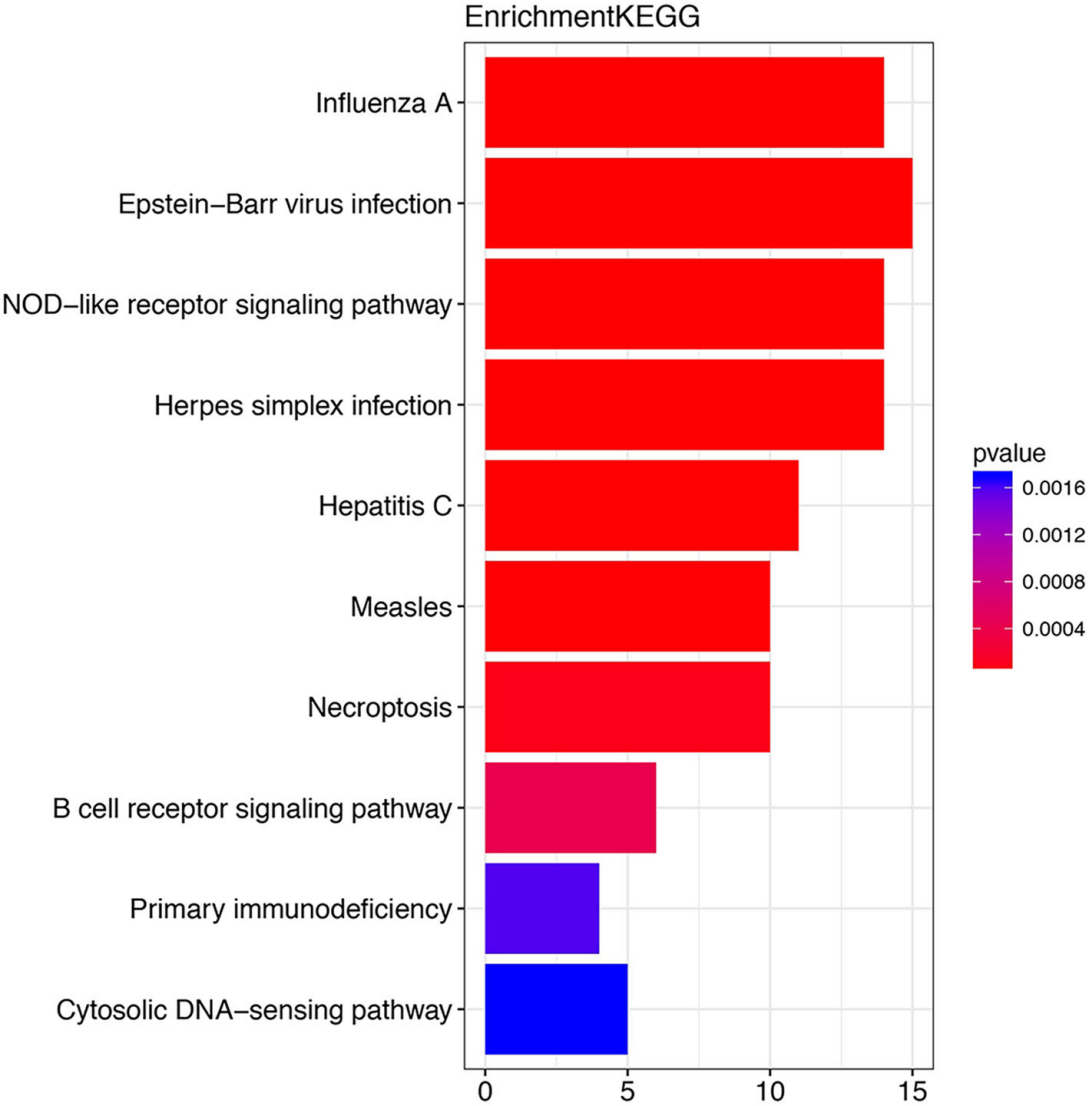
Enrichment analysis for the top five gene sets. We show the top 10 pathways enriched for genes in these five gene sets

**Fig. 6:**
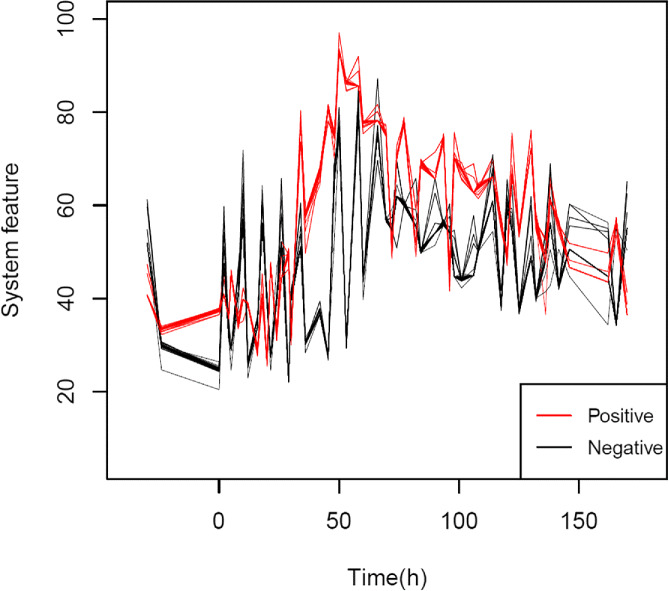
System feature. Dynamic plots of the relationship of genes in the positive and negative groups, respectively. In the early stage (within 24 h), there is a distinction between the positive and negative groups compared with Fig. 3. After the 40th hours, the positive group rises suddenly

The results show that the 44th, 2nd, 34th and 35th gene sets contribute more to the response than the other gene sets. By enrichment analysis for these four gene sets, we can identify pathways related to viruses as shown in Fig. 5. It can be seen that the top pathways are associated with viruses. Finally, we visualize the gene sets associated with response in Fig. 6. Take the positive group as an example. In Fig. 6 each red line represents the change over time of the systematic feature of the gene expression values collected from randomly selecting 80% subjects from the positive group. That is to say, every point on the red line corresponds to the spectral norm of the corresponding matrix of the co-expression network constructed by the genes from the 35th gene set at a certain time *t*. The result shows that there is a clear difference between the positive and negative groups. More importantly, at the early stage, it is very difficult to distinguish the positive and negative groups from the trajectory of a single gene, as shown in Fig. 3. However that is more obvious in Fig. 6, which substantiates our hypothesis that the label of response is related to the dynamic nature of the changing of a system (gene set) but not a single gene. We visualized the co-expression networks of the 35th gene set at time points 0, 12, 24, 48, 96 and 146, respectively [see Additional file 1]. It seems that the connections of gene modules became closer after the samples were exposed to the virus.

## Discussion

In this paper, we adopt a screening approach to find potential gene sets which may be related to response. For this screening step, we do not consider multiple testing when we detect change points of the dynamic networks. We further identify the gene sets related to the response through the proposed Bayesian model. The screening step can be considered as a variable selection step where no response information is used. In addition, when there is no simple relationship between the clinical response and a single gene or a gene set (therefore it is challenging to have statistically significant results for marginal analysis), a model that studies the changes of the relationships among genes in gene sets may offer novel biological insights.

## Conclusions

We have proposed a novel approach of modeling time-series gene expression data for inferring an individual’s response to viral exposure. The biological hypothesis in this paper is that the dynamic changes of the system are related to the clinical response. Compared with previous time series analysis methods, we showed that change point detection for dynamic networks may be informative for the relationship between the clinical response and dynamic nature of the system (gene sets). Joint consideration of multiple kernels based on gene sets with dynamic network structures not only can predict an individual’s clinical response, but can also help elucidate the biological pathways involved. The effectiveness of the proposed method was demonstrated through the analyses of both simulated and real data.

In this paper, we construct the co-expression networks for the gene sets at each time point separately using Pearson correlation. We note that other methods may be used. For example, we can construct networks incorporating some prior knowledge such as regulatory network at each time point to improve network robustness. Some model-based methods such as TV-DBN [11] can be used to construct dynamic networks. Network reconstruction [12] incorporating the temporal nature of the data may help improve the performance of our model. On the other hand, the selection of matrix similarity may influence the change point detection for the networks. It is worth studying the different methods of change-point estimation for networks in the future. Additionally, we considered the case where the response variable is binary. If the response variable is continuous, we will consider a continuous response in the Bayesian model. In real data analysis, we used Pearson correlation coefficient to define similarity function of kernel. Some other kernel functions can be tried, such as dynamic time warping (*dtw*) which has been applied to gene expression data [20]. In practice, cross-validation method can be carried out to select the optimal kernel function definition when the sample size is sufficiently large. In this paper, when we compute multiple kernels for integrating different dynamic gene sets, there is no consideration about relationship between different kernels. However, different gene sets may have overlapping genes, which may influence the estimation of change point. We will consider the Bayesian integration model with correlation information in the future.

## Methods

**Fig. 7:**
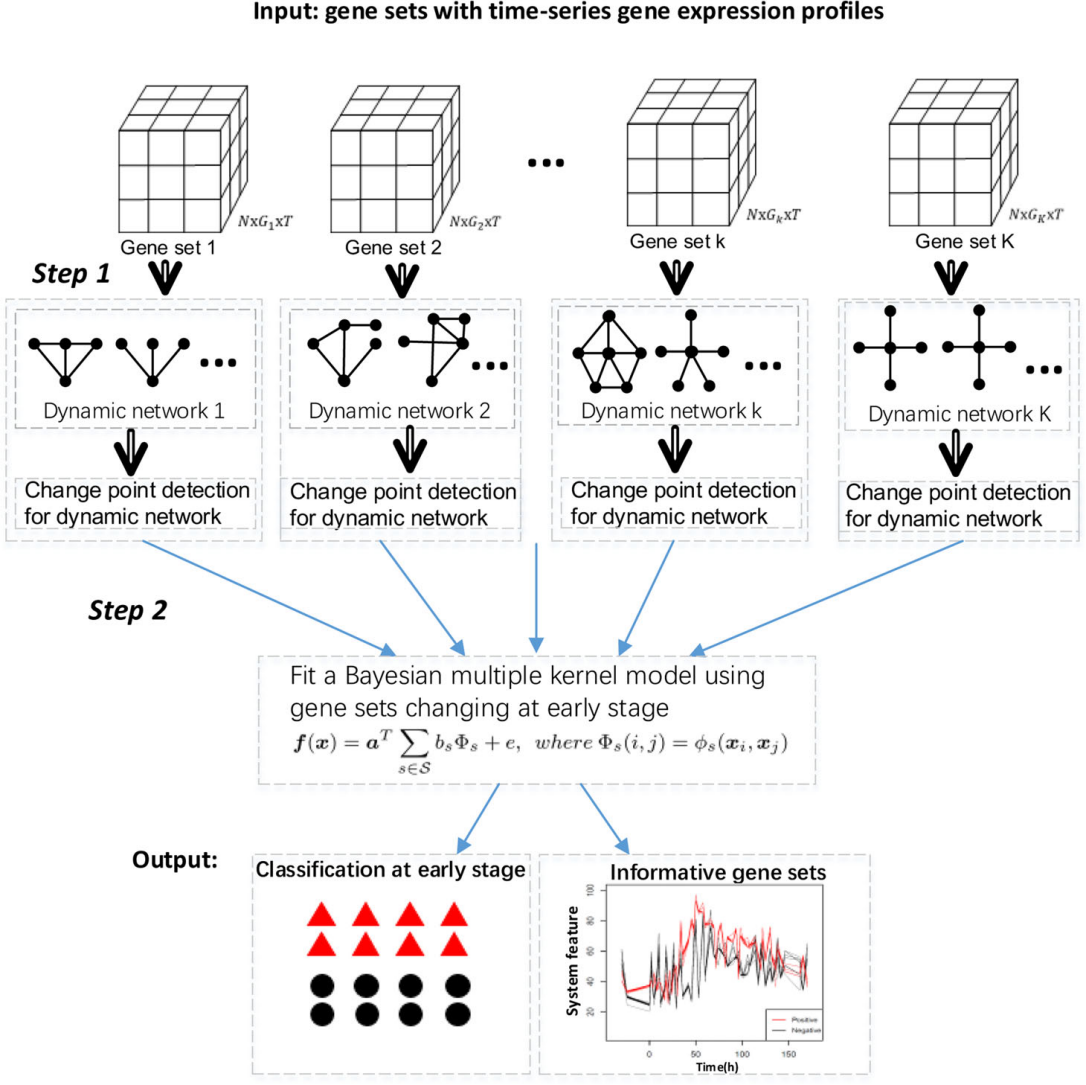
The workflow. Step 1, time-varying networks are constructed for each gene set and change point is detected for these dynamic networks. Step 2, different gene sets which change at early stage are integrated to build a multiple kernel learning model. More details are provided in the “Models” and “Inference” sections

The main aim of the paper is to identify gene sets related to viral exposure response and meanwhile predict a person’s response using the dynamic relationships among genes in a gene set at early exposure stage. We assume that only some of the gene sets are informative about clinical responses. Firstly, the genes need to be organized into different gene sets based on some criteria. Here are some suggested ways to group genes. If there is prior biological knowledge, we can organize genes into different gene sets according to such knowledge. For example, for immune related diseases, the immune-related pathways in the database, MSigDB (http://software.broadinstitute.org/gsea/msigdb/index.jsp) [21], can be viewed as gene sets. Without prior knowledge, we may construct gene sets based on the observed data, e.g. genes with different expression levels at different time points. Secondly, the general framework of our method is summarized in Fig. 7. For each given gene set, time dependent networks are constructed. For example, at each time point, we can construct a fully connected network with genes as nodes and correlation coefficients between genes as weights. Because we hypothesize that the gene sets in which the relationships among genes change over time may be informative about the clinical response, we investigate whether the network has changed over time. The gene sets which change the network structures at early stage are candidate gene sets to predict clinical response. Then, we employ a Bayesian multiple kernel learning model to predict an individual’s response. The key for kernel learning is the definition of similarity. We use the overall relationship between genes to define the similarity between subjects. More details are provided in the “Models” and “Inference” sections.

### Notations

Assume that there are *N* subjects, *G* genes, and *T* time points. Let

- *i* ∈{1,...,*N*} index the subjects,
- *g*$\in \{1,...,G\}\doteq \mathcal {G}$ index the genes,
- *t* ∈{1,...,*T*} index the time points where gene expression data are collected,
- $\boldsymbol {x}\in \mathbb {R}^{\mathrm {N}\times \mathrm {G}\times \mathrm {T}}$ represent the collection of expression values of all genes for all subjects at all time points,
- *y*_*i*_∈{+1,−1} denote the response label of subject *i*,
- $O_{k} \subseteq \mathcal {G}$ be the *k*th subset of gene index set, where *k* is an integer satisfying 1 ≤*k*$\leq 2^{|\mathcal {G}|}-1$ and $|O_{k}|\doteq G_{k}$ and
- $s\in \mathcal {S}$ be the index of kernel, where $\mathcal {S}\subseteq \left \{k: 1\leq k \leq 2^{|\mathcal {G}|}-1\right \}$.

For the elements in the array ***x***, *x*_*igt*_ represents the expression value of gene *g* at time *t* for subject *i*. The data set

$$\boldsymbol{x} = \bigotimes\limits_{i=1}^{N}\bigotimes\limits_{g=1}^{G}\bigotimes\limits_{t=1}^{T}x_{igt}, $$

where $\bigotimes $ represents the Cartesian product. Note that ***x***_*ig*·_=(*x*_*ig*1_,...,*x*_*igT*_)^T^ is the time-series expression observation with length *T* of gene *g* for subject *i*. Similarly, ***x***_*i*·*t*_=(*x*_*i*1*t*_,...,*x*_*iGt*_)^T^ and ***x***_·*gt*_=(*x*_1*gt*_,...,*x*_*Ngt*_)^T^.

### Models

For the *k*th gene set, the genes are collected in *O*_*k*_ and let *G*_*k*_ denote the number of genes in this set. At each time point, we can construct a network such as co-expression network, for genes in the set. Therefore, we can get *T* networks across the *T* time points, with these networks represented by *T* matrices {*A*_1_,...,*A*_*T*_}, where *A*_*t*_(*i*,*j*), the (*i*,*j*)th entry of matrix *A*_*t*_, is derived from *h*(***x***_·*it*_,***x***_·*jt*_), *i*,*j*∈*O*_*k*_ and *h* is a function that defines the correlation or similarity between two genes in this set. The change point detection across these networks can be expressed as follow:

$$H_{0}: A_{t}\sim F_{0} ~~~for ~~1 \leq t \leq T, $$

*vs*

$$H_{1}: \exists \tau, ~where ~1 \leq \tau < T, ~~s.t.~~A_{t}\sim \left\{ \begin{array}{ll} F_{0}, ~~~for ~~1 \leq t \leq \tau, & \\ F_{1}, ~~~for ~~\tau < t \leq T, \end{array}\right. $$

where *F*_0_ and *F*_1_ are different probability measures on a nonzero measure set. Firstly, define the similarity between two matrices as

$$m\left(A_{t_{1}},A_{t_{2}}\right)= \left\|A_{t_{1}}-A_{t_{2}}\right\|_{2},~~~~for ~\forall ~t_{1}, ~t_{2}, $$

where ∥·∥_2_ is the spectral norm of a matrix [22]. The reason for using spectral norm to measure the similarity between two matrices is, for a symmetric matrix, the spectral norm equals to the spectral radius of this symmetric matrix. From a geometric point of view, the spectral radius of a matrix represents the degree of stretching along its corresponding direction. Secondly, we can construct a graph on {*A*_*t*_:*t*=1,...,*T*}, i.e. the minimum spanning tree (MST), with the above definition of matrix similarity. Thirdly, we can detect the change point of {*A*_*t*_:*t*=1,...,*T*}. We use the graph-based change point detection method [23] for statistical inference. More details about change point detection are provided in the Supplementary Materials [see Additional file 1]. We retain the gene sets that change at early stage to build predictive models.

After identifying gene sets with early change points, we use a Bayesian model to integrate dynamic information from multiple gene sets. Assume that the indices of the selected gene sets are collected in $\mathcal {S}$. Each gene set indexed by *s* can define a kernel matrix *Φ*_*s*_. Denote the kernel matrix set $\Phi =\left \{\Phi _{s}: s\in \mathcal {S} \right \}$. We integrate all the $|\mathcal {S}|$ gene sets by the following multiple kernel learning model [24],

1 $$ f\left(\boldsymbol{x}_{i}\right)=\boldsymbol{a}^{\mathrm{T}}\sum_{s\in\mathcal{S}} b_{s} \Phi_{s}^{i} + e,  $$

where ***a***=(*a*_1_,*a*_2_,...,*a*_*N*_)^T^ is the sample weight vector, ***b*** is the kernel weight vector, *e* is bias and $\Phi _{s}^{i}$ is the kernel vector which is the *i*th column of kernel matrix *Φ*_*s*_. The (*i*,*j*)th element of *Φ*_*s*_ is defined by the similarity between subjects *i* and *j*. *Φ*_*s*_(*i*,*j*)=*ϕ*_*s*_(***x***_*i*_,***x***_*j*_), where the kernel function *ϕ*_*s*_ is defined as

$$\phi_{s}\left(\boldsymbol{x}_{i},\boldsymbol{x}_{j}\right)= \left\|\tilde{\Sigma}_{i}^{s}-\tilde{\Sigma}_{j}^{s}\right\|_{2}, ~~\forall i,~j\in\{1,2,...,N\}~and~s\in\mathcal{S}. $$

The (*l*,*k*)th entry of the matrix $\tilde {\Sigma }_{i}^{s}$ is,

$$\tilde{\Sigma}_{i}^{s}(l,k)= \text{cov}\left(\boldsymbol{x}^{s}_{il\cdot},\boldsymbol{x}^{s}_{ik\cdot}\right), ~~~for~l,k\in O_{s}~and~s\in\mathcal{S}, $$

where $\boldsymbol {x}^{s}_{il\cdot }$ represents the expression vector of gene *l* in gene set *O*_*s*_ for subject *i*. In Eq. (1), ***f*** can be considered as a latent variable [25] connecting the observed expression data ***x*** and labels ***y***. Through the estimation of parameter ***b***, we can infer which gene sets have more contribution to the response label ***y***.

### Inference

The main aim of this section is to infer the parameters {***a***,***b***,*e*} in model (1). We adopt a Bayesian framework because of two advantages. Firstly, compared with general kernel-based methods [26, 27], kernel learning under a Bayesian framework reduces the requirement of kernel conditions, such as Mercer’s kernel condition [28, 29]. So we can select more flexible metrics to measure the similarity between subjects based on time series observations. Secondly, compared with general machine learning algorithms, such as SVMs, auxiliary parameters can also be inferred under a Bayesian framework [29]. Denote the priors {***λ***,***γ***,*ω*} corresponding to {***a***,***b***,*e*}, respectively. For computational convenience, we assume conjugate prior distributions [24] in the model. Let ***Ξ***={*α*_*λ*_,*β*_*λ*_,*α*_*γ*_,*β*_*γ*_,*α*_*ω*_,*β*_*ω*_} denote the hyper-parameter set for {***λ***,***γ***,*ω*} and ***L*** be an intermediate output variable for the iteration of parameters. All priors and parameters in the model are denoted by $\boldsymbol {\Theta }=\left \{\boldsymbol {\lambda },\boldsymbol {\gamma },\omega \right \}\bigcup \left \{\boldsymbol {a},\boldsymbol {b},e,\boldsymbol {f},\boldsymbol {L}\right \}$. Hence, the conjugate Bayesian priors for the parameters are

$$\begin{array}{*{20}l} \lambda\lambda_{i} \sim Gamma\left(\lambda_{i};\alpha_{\lambda},\beta_{\lambda}\right) \quad \quad \forall i,\\ a_{i} | \lambda_{i} \sim \mathcal{N}\left(a_{i};0,\lambda_{i}^{-1}\right) \quad \quad \forall i,\\ L_{{si}}| \boldsymbol{a},{\Phi}_{s}^{i}\sim\mathcal{N}\left(L_{{si}};\boldsymbol{a}^{\mathrm{T}}{\Phi_{s}^{i},1} \right) \quad \quad \forall (s,i),\\ \gamma_{s} \sim Gamma\left(\gamma_{s};\alpha_{\gamma},\beta_{\gamma}\right) \quad \quad \forall s,\\ b_{s} | \gamma_{s} \sim \mathcal{N}\left(b_{s};0,\gamma_{s}^{-1}\right) \quad \quad \forall s,\\ \omega \sim Gamma\left(\omega;\alpha_{\omega},\beta_{\omega}\right),\\ e | \omega \sim \mathcal{N}\left(e;0,\omega^{-1}\right),\\ f_{i} | \boldsymbol{b},e,{L}_{\cdot i} \sim \mathcal{N}\left(f_{i};\boldsymbol{b}^{\mathrm{T}{L}}_{\cdot i}+e,1\right) \quad \quad \forall i,\\ y_{i} | f_{i} \sim \delta\left(f_{i}y_{i}>\nu\right) \quad \quad \forall i, \end{array} $$

where *δ*(·) is the Kronecker delta function that returns 1 if the variable satisfies the restriction and 0 otherwise, and *ν* is a given margin parameter which is used to distinguish two categories. Next, we use variational approximation [30, chap.10] to estimate the parameters. The main idea of the algorithm is to approximate the marginal likelihood log*p*(***y***|***x***) by the lower bound $\mathcal {L}$,

$$\log p\left(\boldsymbol{y}|\boldsymbol{x}\right)\geq \mathcal{L} \doteq E_{q(\boldsymbol{\Theta})}\left[\log p\left(\boldsymbol{y},\boldsymbol{\Theta}|\boldsymbol{x}\right)\right]-E_{q(\boldsymbol{\Theta})}\left[\log q(\boldsymbol{\Theta})\right], $$

where E represents the expectation of random variables and *q*(***Θ***) is the posterior distribution of ***Θ***. The exact formulas of the lower bound $\mathcal {L}$ are similar to those in the supplementary material of reference [24]. Hence, the approximate posterior distribution *q*(·) of each parameter can be computed by

2 $$ q(\cdot) \propto exp\left\{E_{q\left(\boldsymbol{\Theta}\backslash\cdot\right)}\left[\log p\left(\boldsymbol{y},\boldsymbol{\Theta}|\boldsymbol{x}\right)\right]\right\},  $$

where *q*(***Θ***∖·) is the distribution of ***Θ*** with the parameter (·) removed. Algorithm 1 summarizes the estimation process of model parameters {***a***,***b***,*e*,***f***,***L***}. After we obtain a trained model, the label for a new subject can be predicted by Eq. (1). More details about Algorithm 1 can be found in the Supplementary Materials [see Additional file 1].


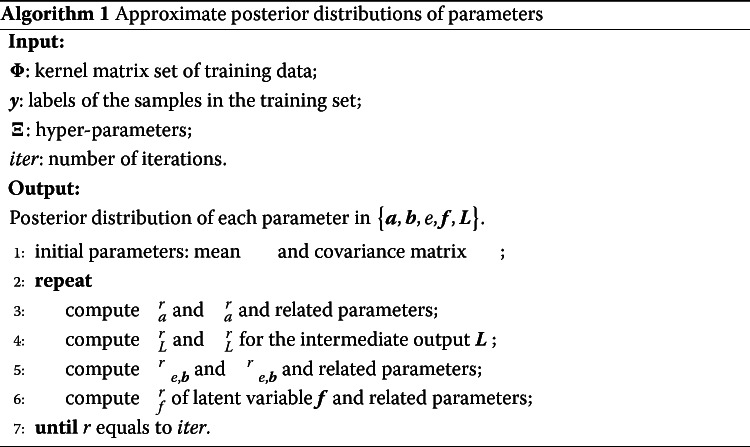


## Supplementary information

**Additional file 1** Supplementary Materials include six sections: Section 1, Graph-based Change-point Detection; Section 2, Details of Algorithm 1; Section 3, More Simulations; Section 4, Analysis of the Effects of Gene Sets; Section 5, Challenge Results; and Section 6, Figures.

**Additional file 2** An example of the R code used in the paper.

## Data Availability

The datasets analysed during the current study are available from the corresponding author on reasonable request. The R code used during this study are included in this published article [see Additional file 2].

## Abbreviations

DBN: Dynamic Bayesian Network
TV-DBN: Time-Varying Dynamic Bayesian Networks
dMMSB: dynamic Mixed Membership Stochastic Block model
M-DMs: Multiple Differential Modules
MSigDB: Molecular Signatures Database
MST: Minimum Spanning Tree
LR: Logistic Regression
LDA: Linear Discriminant Analysis
SVM: Support Vector Machine
KNN: K-Nearest Neighbor
CHP: Change Point Position
ROC: Receiver Operating Characteristic curve
FPR: False Positive Rate
TPR: True Positive Rate
AUC: Area Under Curve
DTW: Dynamic Time Warping
