# Supplementary material for: Predicting viral exposure response from modeling the changes of co-expression networks using time series gene expression data
Source: BMC Bioinformatics. 2020 Aug 26;21:370. doi: 10.1186/s12859-020-03705-0 (PMC7449007; doi:10.1186/s12859-020-03705-0)
Supplement: Supplementary file 1 — Additional file 1 Supplementary Materials include six sections: Section 1, Graph-based Change-point Detection; Section 2, Details of Algorithm 1; Section 3, More Simulations; Section 4, Analysis of the Effects of Gene Sets; Section 5, Challenge Results; and Section 6, Figures. [file 12859_2020_3705_MOESM1_ESM.pdf]

Supplementary Materials

1. Graph-based Change-point Detection

For the hypothesis testing problem in Section 2.2, under H_1, the observations $\{A_t : t = 1, 2, ..., T\}$ are assumed to have distribution F_0 for $t \leq \tau$ and a different distribution F_1 for $t > \tau$. With the definition of matrix similarity $m(A_i, A_j)$, we can construct a similarity graph on $\{A_t\}$ (Chen and Zhang, 2015, Section 2.1). Let E denote the set of edges in the similarity graph. Hence, each possible value of τ can partition the observations into two groups: those observations before τ and those after τ. For each candidate value t of τ, the number of edges connecting points from different groups is

$$R_E(t) = \sum_{(t_1,t_2)\in E} I_{E_{t_1}(t) \neq E_{t_2}(t)}, \quad E_s(t) = I_{s>t}, \ s = t_1, t_2,$$

where (t_1, t_2) is the edge connecting A_{t_1} and A_{t_2}. Here, $E_s(t)$ is an indicator function for the event that A_s is observed after t. $R_E(t)$ is the number of edges in the similarity graph that connect observations from the "past" ($\leq t$) to the "future" ($> t$). Since the null distribution of $R_E(t)$ depends on t, the authors standardize $R_E(t)$ so that it is comparable across t. Let

$$Z_E(t) = -\frac{R_E(t) - \mathbf{E}[R_E(t)]}{\sqrt{\mathbf{Var}[R_E(t)]}}.$$

The mean $\mathbf{E}[R_E(t)]$ and variance $\mathbf{Var}[R_E(t)]$ are computed by LEMMA 2.1 in the paper (Chen and Zhang, 2015). Relatively large values of $Z_E(t)$ are evidence against the null hypothesis. So we use the following scan statistic to test H_0 versus H_1.

$$\max_{n_0 \leq t \leq n_1} Z_E(t),$$

where n_0 and n_1 are prespecified constraints for τ which satisfy $1 < n_0 \leq \tau \leq n_1 < n$ for a single change point. The significance levels are studied in Section 3 of Chen and Zhang (2015).

2. Details of Algorithm 1

In Section 2.2, we denote the kernel matrix set $\mathbf{\Phi} = \{\Phi_s : s \in \mathcal{S}\}$ and integrate all the $|\mathcal{S}|$ gene sets by multiple kernel learning model as,

$$f(\boldsymbol{x}_i) = \boldsymbol{a}^{\mathrm{T}} \sum_{s\in\mathcal{S}} b_s \Phi_s^i + e, \tag{1}$$

where Φ_s^i is the kernel vector which is the ith column of Φ_s. Firstly, we recall the conjugate Bayesian model (Gönen, 2012),

$$\begin{aligned} \lambda_i &\sim Gamma(\lambda_i; \alpha_\lambda, \beta_\lambda) \qquad &\forall i, \\ a_i|\lambda_i &\sim \mathcal{N}(a_i; 0, \lambda_i^{-1}) \qquad &\forall i, \end{aligned}$$

$$
\begin{aligned}
L_{si}|\boldsymbol{a}, \Phi_s^i &\sim \mathcal{N}(L_{si}; \boldsymbol{a}^{\mathrm{T}}\Phi_s^i, 1) && \forall(s,i),\\
\gamma_s &\sim Gamma(\gamma_s; \alpha_\gamma, \beta_\gamma) && \forall s,\\
b_s|\gamma_s &\sim \mathcal{N}(b_s; 0, \gamma_s^{-1}) && \forall s,\\
\omega &\sim Gamma(\omega; \alpha_\omega, \beta_\omega), &&\\
e|\omega &\sim \mathcal{N}(e; 0, \omega^{-1}), &&\\
f_i|\boldsymbol{b}, e, L_{\cdot i} &\sim \mathcal{N}(f_i; \boldsymbol{b}^{\mathrm{T}}L_{\cdot i} + e, 1) && \forall i,\\
y_i|f_i &\sim \delta(f_i y_i > \nu) && \forall i,
\end{aligned}
$$

where $Gamma(\cdot; \alpha, \beta)$ is the gamma distribution with mean $\alpha\beta$ and variance $\alpha\beta^2$. $\mathcal{N}(\cdot; \mu, \Sigma)$ represents multivariate normal distribution with mean μ and covariance Σ. $\delta(\cdot)$ is Kronecker delta function that returns 1 if the variable satisfies the restriction and 0 otherwise. Next, we use variational approximation to estimate the parameters. Hence the approximate posterior distribution of a specific variable is

$$
q(\cdot) \propto exp\{\mathrm{E}_{q(\boldsymbol{\Theta}\backslash\cdot)}[\log p(\boldsymbol{y}, \boldsymbol{\Theta}|\boldsymbol{x})]\}, \tag{2}
$$

where $q(\boldsymbol{\Theta}\backslash\cdot)$ denotes the distribution of the parameter set $\boldsymbol{\Theta}$ with the parameter $(\cdot)$ removed. Under the variational approximation framework (Bishop, 2007, chap.10), we know the likelihood function and factor posterior distribution are

$$
\begin{aligned}
p(\boldsymbol{y}, \boldsymbol{\Theta}|\boldsymbol{\Phi}) &= p(\boldsymbol{\lambda})p(\boldsymbol{a}|\boldsymbol{\lambda})p(L|\boldsymbol{a}, \boldsymbol{\Phi})p(\boldsymbol{\gamma})p(\boldsymbol{b}|\boldsymbol{\gamma})p(\omega)p(e|\omega)p(\boldsymbol{f}|\boldsymbol{b}, e, L)p(\boldsymbol{y}|\boldsymbol{f}),\\
q(\boldsymbol{\Theta}) &= q(\boldsymbol{\lambda})q(\boldsymbol{a})q(L)q(\boldsymbol{\gamma})q(\omega)q(e, \boldsymbol{b})q(\boldsymbol{f}).
\end{aligned}
$$

Hence, through Eq. (2) we can compute the posterior distribution of $\boldsymbol{\lambda}$ as

$$
\begin{aligned}
q(\boldsymbol{\lambda}) &= exp\{\mathrm{E}_{q(\boldsymbol{\Theta}\backslash\boldsymbol{\lambda})}\left[\log p(\boldsymbol{y}, \boldsymbol{\Theta}|\boldsymbol{\Phi})\right]\}\\
&= exp\{\mathrm{E}_{q(\boldsymbol{a})q(L)q(\boldsymbol{\gamma})q(\omega)q(e,\boldsymbol{b})q(\boldsymbol{f})}\left[\log p(\boldsymbol{y}, \boldsymbol{\Theta}|\boldsymbol{\Phi})\right]\}\\
&= exp\{\log p(\boldsymbol{\lambda}) + \mathrm{E}_{q(\boldsymbol{a})}[\log p(\boldsymbol{a}|\boldsymbol{\lambda})] + c_0\}\\
&= exp\left\{\log \prod_{i=1}^{N} p(\lambda_i) + \mathrm{E}_{q(\boldsymbol{a})}\left[\log \prod_{i=1}^{N}\left(\frac{\lambda_i^{1/2}}{\sqrt{2\pi}}e^{-\frac{\lambda_i a_i^2}{2}}\right)\right] + c_0\right\}\\
&= c_1 \prod_{i=1}^{N}\left\{\frac{\beta_\lambda^{-\alpha_\lambda}}{\Gamma(\alpha_\lambda)}e^{-\beta_\lambda^{-1}\lambda_i}\lambda_i^{\alpha_\lambda - 1}\lambda_i^{\frac{1}{2}}e^{-\frac{\lambda_i}{2}Ea_i^2}\right\}\\
&= c_2 \prod_{i=1}^{N}\left\{\lambda_i^{(\alpha_\lambda+\frac{1}{2})-1}e^{-\left(\beta_\lambda^{-1}+\frac{Ea_i^2}{2}\right)\lambda_i}\right\},
\end{aligned}
$$

where c_0, c_1 and c_2 are constants. Hence, the posterior distribution of $\boldsymbol{\lambda}$ is summarized as

$$
q(\boldsymbol{\lambda}) = \prod_{i=1}^{N} Gamma\left(\lambda_i; \alpha_\lambda + \frac{1}{2}, \left(\frac{1}{\beta_\lambda} + \frac{\mathrm{E}a_i^2}{2}\right)^{-1}\right).
$$

Similarly, we can compute $q(\boldsymbol{\gamma})$ and $q(\omega)$ as

$$q(\boldsymbol{\gamma}) = \prod_{s=1}^{|\mathcal{S}|} Gamma\left(\gamma_s; \alpha_\gamma + \frac{1}{2}, \left(\frac{1}{\beta_\gamma} + \frac{\mathrm{E}b_s^2}{2}\right)^{-1}\right),$$

$$q(\omega) = Gamma\left(\omega; \alpha_\omega + \frac{1}{2}, \left(\frac{1}{\beta_\omega} + \frac{\mathrm{E}e^2}{2}\right)^{-1}\right).$$

The approximate posterior distribution of $\boldsymbol{a}$ is calculated as

$$\begin{aligned}
q(\boldsymbol{a}) &= exp\{\mathrm{E}_{q(\boldsymbol{\Theta}\backslash\boldsymbol{a})}[\log p(\boldsymbol{y}, \boldsymbol{\Theta}|\boldsymbol{\Phi})]\} \\
&= exp\{\mathrm{E}_{q(\boldsymbol{\lambda})}[\log p(\boldsymbol{a}|\boldsymbol{\lambda})] + \mathrm{E}_{q(L)}[\log p(L|\boldsymbol{a}, \boldsymbol{\Phi})]\} \\
&= exp\Bigg\{\mathrm{E}_{q(\boldsymbol{\lambda})}\left[-\frac{1}{2}\boldsymbol{a}^{\mathrm{T}}\mathrm{diag}(\boldsymbol{\lambda})\boldsymbol{a}\right] \\
&\quad + \mathrm{E}_{q(L)}\left[\log \prod_{s=1}^{|\mathcal{S}|} exp\left(-\frac{1}{2}(L_{s\cdot}^{\mathrm{T}} - (\boldsymbol{a}^{\mathrm{T}}\Phi_s)^{\mathrm{T}})^{\mathrm{T}}(L_{s\cdot}^{\mathrm{T}} - (\boldsymbol{a}^{\mathrm{T}}\Phi_s)^{\mathrm{T}})\right)\right]\Bigg\} \\
&= exp\Bigg\{-\frac{1}{2}\Bigg[\boldsymbol{a}^{\mathrm{T}}\left(\mathrm{diag}(\mathrm{E}\boldsymbol{\lambda}) + \sum_{s=1}^{|\mathcal{S}|}\Phi_s\Phi_s^{\mathrm{T}}\right)\boldsymbol{a} - \sum_{s=1}^{|\mathcal{S}|}\mathrm{E}L_{s\cdot}\Phi_s^{\mathrm{T}}\boldsymbol{a} \\
&\quad - \sum_{s=1}^{|\mathcal{S}|}\boldsymbol{a}^{\mathrm{T}}\Phi_s\mathrm{E}L_{s\cdot}^{\mathrm{T}}\Bigg]\Bigg\}.
\end{aligned}$$

Let

$$\Sigma_a^{-1} = \mathrm{diag}(\mathrm{E}\boldsymbol{\lambda}) + \sum_{s=1}^{|\mathcal{S}|}\Phi_s\Phi_s^{\mathrm{T}},$$

and

$$\Sigma_a^{-1}\mu_a = \sum_{s=1}^{|\mathcal{S}|}\Phi_s\mathrm{E}L_{s\cdot}^{\mathrm{T}}, \quad \mu_a = \Sigma_a\sum_{s=1}^{|\mathcal{S}|}\Phi_s\mathrm{E}L_{s\cdot}^{\mathrm{T}}.$$

Hence, the posterior distribution of $\boldsymbol{a}$ is

$$q(\boldsymbol{a}) = \mathcal{N}\left(\boldsymbol{a}; \Sigma_a\sum_{s=1}^{|\mathcal{S}|}\Phi_s\mathrm{E}L_{s\cdot}^{\mathrm{T}}, \left(\mathrm{diag}(\mathrm{E}\boldsymbol{\lambda}) + \sum_{s=1}^{|\mathcal{S}|}\Phi_s\Phi_s^{\mathrm{T}}\right)^{-1}\right).$$

The posterior distribution of $(e, \boldsymbol{b})$ is calculated similarly as

$$
\begin{aligned}
q(e, \boldsymbol{b}) &= exp\{\mathrm{E}_{q(\boldsymbol{\Theta}\backslash e,\boldsymbol{b})}[\log p(\boldsymbol{y}, \boldsymbol{\Theta}|\boldsymbol{\Phi})]\} \\
&= exp\{\mathrm{E}_{q(\boldsymbol{\gamma})q(\omega)q(\boldsymbol{f})q(L)}[\log p(\boldsymbol{b}|\boldsymbol{\gamma})p(e|\omega)p(\boldsymbol{f}|\boldsymbol{b}, e, L)]\} \\
&= exp\{\mathrm{E}_{q(\boldsymbol{\gamma})}[\log p(\boldsymbol{b}|\boldsymbol{\gamma})] + \mathrm{E}_{q(\omega)}[\log p(e|\omega)] + \mathrm{E}_{q(\boldsymbol{f})q(L)}[\log p(\boldsymbol{f}|\boldsymbol{b}, e, L)]\} \\
&= exp\Bigg\{\mathrm{E}_{q(\boldsymbol{\gamma})}\left[\log exp\left(-\frac{1}{2}\boldsymbol{b}^{\mathrm{T}}\mathrm{diag}(\boldsymbol{\gamma})\boldsymbol{b}\right)\right] + \mathrm{E}_{q(\omega)}\left[\log exp\left(-\frac{1}{2}e^{\mathrm{T}}\omega e\right)\right] \\
&\quad + \mathrm{E}_{q(\boldsymbol{f})q(L)}\left[\log exp\left(-\frac{1}{2}(\boldsymbol{f} - (\boldsymbol{b}^{\mathrm{T}}L)^{\mathrm{T}} - e\mathbf{1})^{\mathrm{T}}(\boldsymbol{f} - (\boldsymbol{b}^{\mathrm{T}}L)^{\mathrm{T}} - e\mathbf{1})\right)\right]\Bigg\} \\
&= exp\Bigg\{-\frac{1}{2}\Bigg[\begin{pmatrix} e \\ \boldsymbol{b} \end{pmatrix}^{\mathrm{T}}\begin{pmatrix} \mathrm{E}\omega + \mathbf{1}^{\mathrm{T}}\mathbf{1} & \mathbf{1}^{\mathrm{T}}\mathrm{E}L^{\mathrm{T}} \\ \mathrm{E}L\mathbf{1} & \mathrm{diag}(\mathrm{E}\boldsymbol{\gamma}) + \mathrm{E}LL^{\mathrm{T}} \end{pmatrix}\begin{pmatrix} e \\ \boldsymbol{b} \end{pmatrix} \\
&\quad - \begin{pmatrix} e \\ \boldsymbol{b} \end{pmatrix}^{\mathrm{T}}\begin{pmatrix} \mathbf{1}^{\mathrm{T}}\mathrm{E}\boldsymbol{f} \\ \mathrm{E}L\mathrm{E}\boldsymbol{f} \end{pmatrix} - \begin{pmatrix} \mathbf{1}^{\mathrm{T}}\mathrm{E}\boldsymbol{f} \\ \mathrm{E}L\mathrm{E}\boldsymbol{f} \end{pmatrix}^{\mathrm{T}}\begin{pmatrix} e \\ \boldsymbol{b} \end{pmatrix}\Bigg]\Bigg\}.
\end{aligned}
$$

Hence, we obtain the posterior distribution of $(e, \boldsymbol{b})$ as

$$
q(e, \boldsymbol{b}) = \mathcal{N}\left(\begin{pmatrix} e \\ \boldsymbol{b} \end{pmatrix}; \Sigma_{(e,\boldsymbol{b})}\begin{pmatrix} \mathbf{1}^{\mathrm{T}}\mathrm{E}\boldsymbol{f} \\ \mathrm{E}L\mathrm{E}\boldsymbol{f} \end{pmatrix}, \begin{pmatrix} \mathrm{E}\omega + N & \mathbf{1}^{\mathrm{T}}\mathrm{E}L^{\mathrm{T}} \\ \mathrm{E}L\mathbf{1} & \mathrm{diag}(\mathrm{E}\boldsymbol{\gamma}) + \mathrm{E}LL^{\mathrm{T}} \end{pmatrix}^{-1}\right).
$$

Next, we update the intermediate variable L as follows:

$$
\begin{aligned}
q(L) &= exp\{\mathrm{E}_{q(\boldsymbol{\Theta}\backslash L)}[\log p(\boldsymbol{y}, \boldsymbol{\Theta}|\boldsymbol{\Phi})]\} \\
&= exp\{\mathrm{E}_{q(\boldsymbol{a})q(\boldsymbol{f})q(e,\boldsymbol{b})}[\log p(L|\boldsymbol{a}, \boldsymbol{\Phi})p(\boldsymbol{f}|\boldsymbol{b}, e, L)]\} \\
&= exp\Bigg\{\mathrm{E}_{q(\boldsymbol{a})}\left[\log\prod_{i=1}^{N} exp\left(-\frac{1}{2}(L_{\cdot i} - \mathcal{K}\boldsymbol{a})^{\mathrm{T}}(L_{\cdot i} - \mathcal{K}\boldsymbol{a})\right)\right] \\
&\quad + \mathrm{E}_{q(\boldsymbol{f})q(e,\boldsymbol{b})}\left[\log\prod_{i=1}^{N} exp\left(-\frac{1}{2}(f_i - \boldsymbol{b}^{\mathrm{T}}L_{\cdot i} - e)^{\mathrm{T}}(f_i - \boldsymbol{b}^{\mathrm{T}}L_{\cdot i} - e)\right)\right]\Bigg\},
\end{aligned}
$$

where $\mathcal{K} \triangleq (\Phi_1^i, \cdots, \Phi_m^i)^{\mathrm{T}}$ and $m \triangleq |\ \mathcal{S}\ |$. We obtain the formula

$$
\begin{aligned}
q(L) = exp\Bigg\{&\sum_{i=1}^{N}\mathrm{E}_{q(\boldsymbol{a})}\left[-\frac{1}{2}(L_{\cdot i}^{\mathrm{T}}L_{\cdot i} - L_{\cdot i}^{\mathrm{T}}\mathcal{K}\boldsymbol{a} - \boldsymbol{a}^{\mathrm{T}}\mathcal{K}^{\mathrm{T}}L_{\cdot i})\right] \\
&+ \sum_{i=1}^{N}\mathrm{E}_{q(\boldsymbol{f})q(e,\boldsymbol{b})}\left[-\frac{1}{2}(-f_i^{\mathrm{T}}\boldsymbol{b}^{\mathrm{T}}L_{\cdot i} - L_{\cdot i}^{\mathrm{T}}\boldsymbol{b}f_i + L_{\cdot i}^{\mathrm{T}}\boldsymbol{b}\boldsymbol{b}^{\mathrm{T}}L_{\cdot i} + L_{\cdot i}^{\mathrm{T}}\boldsymbol{b}e + e^{\mathrm{T}}\boldsymbol{b}^{\mathrm{T}}L_{\cdot i})\right]\Bigg\}.
\end{aligned}
$$

Denote $\Sigma_L^{-1} = \mathbb{I} + \mathrm{E}\boldsymbol{b}\boldsymbol{b}^{\mathrm{T}}$, then

$$
\Sigma_L^{-1}\mu_{L_{\cdot i}} = \mathcal{K}\mathrm{E}\boldsymbol{a} + \mathrm{E}\boldsymbol{b}\mathrm{E}f_i - \mathrm{E}\boldsymbol{b}e, \quad \mu_{L_{\cdot i}} = \Sigma_L(\mathcal{K}\mathrm{E}\boldsymbol{a} + \mathrm{E}\boldsymbol{b}\mathrm{E}f_i - \mathrm{E}\boldsymbol{b}e).
$$

We obtain the approximate posterior distribution of intermediate variable L as

$$q(L) = \prod_{i=1}^{N} \mathcal{N}\left(L_{\cdot i}; \Sigma_L \left[\begin{pmatrix} (\Phi_1^i)^{\mathrm{T}} \\ \vdots \\ (\Phi_m^i)^{\mathrm{T}} \end{pmatrix} \mathrm{E}\boldsymbol{a} + \mathrm{E}\boldsymbol{b}\mathrm{E}f_i - \mathrm{E}\boldsymbol{b}e\right], \left(\mathbb{I} + \mathrm{E}\boldsymbol{b}\boldsymbol{b}^{\mathrm{T}}\right)^{-1}\right).$$

After we have the approximate posterior distributions of $(e, \boldsymbol{b})$ and L, we can compute the posterior distribution of the latent variable $\boldsymbol{f}$. Therefore, the posterior distribution of $\boldsymbol{f}$ is

$$q(\boldsymbol{f}) = \prod_{i=1}^{N} \mathcal{TN}\left(f_i; \mathrm{E}\boldsymbol{b}^{\mathrm{T}}\mathrm{E}L_{\cdot i} + \mathrm{E}e, 1, f_i y_i > \nu\right).$$

The approximate posterior distributions of parameters can be summarized as:

$$\begin{aligned}
q(\boldsymbol{\lambda}) &= \prod_{i=1}^{N} Gamma\left(\lambda_i; \alpha_\lambda + \frac{1}{2}, \left(\frac{1}{\beta_\lambda} + \frac{\widetilde{a_i^2}}{2}\right)^{-1}\right), \\
q(\boldsymbol{a}) &= \mathcal{N}\left(\boldsymbol{a}; \Sigma_a \sum_{s=1}^{|\mathcal{S}|} \Phi_s \widetilde{L_{s\cdot}^{\mathrm{T}}}, \left(\mathrm{diag}(\tilde{\boldsymbol{\lambda}}) + \sum_{s=1}^{|\mathcal{S}|} \Phi_s \Phi_s^{\mathrm{T}}\right)^{-1}\right), \\
q(L) &= \prod_{i=1}^{N} \mathcal{N}\left(L_{\cdot i}; \Sigma_L \left[\begin{pmatrix} (\Phi_1^i)^{\mathrm{T}} \\ \vdots \\ (\Phi_m^i)^{\mathrm{T}} \end{pmatrix} \tilde{\boldsymbol{a}} + \tilde{\boldsymbol{b}}\tilde{f}_i - \widetilde{\boldsymbol{b}e}\right], \left(\mathbb{I} + \widetilde{\boldsymbol{b}\boldsymbol{b}^{\mathrm{T}}}\right)^{-1}\right), \\
q(\boldsymbol{\gamma}) &= \prod_{s=1}^{|\mathcal{S}|} Gamma\left(\gamma_s; \alpha_\gamma + \frac{1}{2}, \left(\frac{1}{\beta_\gamma} + \frac{\widetilde{b_s^2}}{2}\right)^{-1}\right), \\
q(\omega) &= Gamma\left(\omega; \alpha_\omega + \frac{1}{2}, \left(\frac{1}{\beta_\omega} + \frac{\widetilde{e^2}}{2}\right)^{-1}\right), \\
q(e, \boldsymbol{b}) &= \mathcal{N}\left(\begin{pmatrix} e \\ \boldsymbol{b} \end{pmatrix}; \Sigma_{(e,\boldsymbol{b})} \begin{pmatrix} \mathbf{1}^{\mathrm{T}}\tilde{\boldsymbol{f}} \\ \tilde{L}\tilde{\boldsymbol{f}} \end{pmatrix}, \begin{pmatrix} \tilde{\omega} + N & \mathbf{1}^{\mathrm{T}}\widetilde{L^{\mathrm{T}}} \\ \tilde{L}\mathbf{1} & \mathrm{diag}(\tilde{\boldsymbol{\gamma}}) + \widetilde{LL^{\mathrm{T}}} \end{pmatrix}^{-1}\right), \\
q(\boldsymbol{f}) &= \prod_{i=1}^{N} \mathcal{TN}\left(f_i; \widetilde{\boldsymbol{b}^{\mathrm{T}}}\widetilde{L_{\cdot i}} + \tilde{e}, 1, f_i y_i > \nu\right),
\end{aligned}$$

where $\widetilde{(\cdot)}$ represents the expectation operator, $\mathbb{I}$ is the identity matrix, $\mathbf{1}$ is a column vector of 1, and $m = |\mathcal{S}|$ is the total number of selected gene sets by change point detection. Hence we can use the posterior distributions of parameters $\{\boldsymbol{a}, \boldsymbol{b}, e, \boldsymbol{f}, \boldsymbol{L}\}$ for inference. Denote $\mathrm{diag}(\cdot)$ the diagonal matrix with diagonal elements $(\cdot)$. So the iterative formulas in Algorithm 1 are

$$
\begin{aligned}
&(\Sigma_a^r)^{-1} = \mathrm{diag}(\mathrm{E}\boldsymbol{\lambda}) + \sum_{s\in\mathcal{S}} \Phi_s\Phi_s^{\mathrm{T}}, \quad \mu_a^r = \Sigma_a \sum_{s\in\mathcal{S}} \Phi_s \mathrm{E}(L_{s\cdot})^{\mathrm{T}},\\
&(\Sigma_L^r)^{-1} = \mathbb{I} + \mathrm{E}\boldsymbol{b}\boldsymbol{b}^{\mathrm{T}}, \quad \mu_{L\cdot i}^r = \Sigma_L \left[\begin{pmatrix} (\Phi_1^i)^{\mathrm{T}} \\ \vdots \\ (\Phi_{|\mathcal{S}|}^i)^{\mathrm{T}} \end{pmatrix} \mathrm{E}\boldsymbol{a} + \mathrm{E}\boldsymbol{b}\mathrm{E}f_i - \mathrm{E}(e\boldsymbol{b})\right],\\
&(\Sigma_{(e,\boldsymbol{b})}^r)^{-1} = \begin{pmatrix} \mathrm{E}\omega + N & \mathbf{1}^{\mathrm{T}}\mathrm{E}L^{\mathrm{T}} \\ \mathrm{E}L\mathbf{1} & \mathrm{diag}(\mathrm{E}\boldsymbol{\gamma}) + \mathrm{E}LL^{\mathrm{T}} \end{pmatrix}, \quad \mu_{(e,\boldsymbol{b})}^r = \Sigma_{(e,\boldsymbol{b})} \begin{pmatrix} \mathbf{1}^{\mathrm{T}}\mathrm{E}\boldsymbol{f} \\ \mathrm{E}L\mathrm{E}\boldsymbol{f} \end{pmatrix},\\
&q^r(\boldsymbol{f}) = \prod_{i=1}^{N} \mathcal{TN}\left(f_i; \mathrm{E}\boldsymbol{b}^{\mathrm{T}}\mathrm{E}L_{\cdot i} + \mathrm{E}e, 1, f_i y_i > \nu\right),
\end{aligned}
$$

where E represents the expectation of random variables.

After we obtain the trained model above, the label for a new subject $\boldsymbol{x}_*$ can be predicted by Eq. (1). Firstly, we can get the distribution of $L_{\cdot *}$ as

$$p(L_{\cdot *}|\boldsymbol{\Phi}, \boldsymbol{a}, \boldsymbol{\Phi}^*) = \prod_{s=1}^{|\mathcal{S}|} \mathcal{N}\left(L_{s*}; \mu^{\mathrm{T}}(\boldsymbol{a})\Phi_s^*, 1 + (\Phi_s^*)^{\mathrm{T}}\Sigma(\boldsymbol{a})\Phi_s^*\right).$$

Secondly, the distribution of f_* can be computed by the following formula as

$$p(f_*|L_{\cdot *}, \boldsymbol{b}, e) = \mathcal{N}\left(f_*; \mu^{\mathrm{T}}(e, \boldsymbol{b}) \begin{pmatrix} 1 \\ L_{\cdot *} \end{pmatrix}, 1 + \begin{pmatrix} 1 & L_{\cdot *}^{\mathrm{T}} \end{pmatrix} \Sigma(e, \boldsymbol{b}) \begin{pmatrix} 1 \\ L_{\cdot *} \end{pmatrix}\right).$$

Hence, the predictive distribution of the label y_* for a new instance $\boldsymbol{x}_*$ is

$$
\begin{aligned}
p(y_* = +1|\boldsymbol{\Phi}^*, \boldsymbol{\Phi}, \boldsymbol{y}) &= Z_*^{-1}\Phi(\frac{\mu(f_*) - \nu}{\Sigma(f_*)}),\\
p(y_* = -1|\boldsymbol{\Phi}^*, \boldsymbol{\Phi}, \boldsymbol{y}) &= Z_*^{-1}\Phi(\frac{-\mu(f_*) - \nu}{\Sigma(f_*)}),
\end{aligned}
$$

where $Z_* = \Phi(\frac{\mu(f_*)-\nu}{\Sigma(f_*)}) + \Phi(\frac{-\mu(f_*)-\nu}{\Sigma(f_*)})$ is the normalization coefficient and here $\Phi(\cdot)$ is the standardized normal cumulative distribution function.

3. More Simulations

In this section, we show the evaluation results of our methods in the cases $(N, T) \in \{(20, 40), (20, 80), (20, 150), (50, 40), (50, 80), (50, 150), (100, 80), (100, 150)\}$. To simplify the calculation, we fixed $G = 80$ and $\rho = 0.7$. The time-series gene expression data are simulated for $N/2$ subjects labelled '+1' through the model,

- $\boldsymbol{x}_{iO_1t_1} \sim \mathcal{N}(\boldsymbol{\mu}_t, \Sigma_0), \quad i = 1, ..., N/2, \; t_1 = 1, ..., T/2,$
  $\boldsymbol{x}_{iO_1t_2} \sim \mathcal{N}(\boldsymbol{\mu}_t, \Sigma_1), \quad i = 1, ..., N/2, \; t_2 = (T/2 + 1), ..., T,$
- $\boldsymbol{x}_{iO_2t} \sim \mathcal{N}(\boldsymbol{\mu}_t, \Sigma_0), \quad i = 1, ..., N/2, \; t = 1, ..., T,$

- $\boldsymbol{x}_{iO_3t_1} \sim \mathcal{N}(\boldsymbol{\mu}_t, \Sigma_1), \quad i = 1, ..., N/2, \ t_1 = 1, ..., T/2,$
  $\boldsymbol{x}_{iO_3t_2} \sim \mathcal{N}(\boldsymbol{\mu}_t, \Sigma_0), \quad i = 1, ..., N/2, \ t_2 = (T/2+1), ..., T,$
- $\boldsymbol{x}_{iO_4t} \sim \mathcal{N}(\boldsymbol{\mu}_t, \Sigma_1), \quad i = 1, ..., N/2, \ t = 1, ..., T.$

For $N/2$ subjects labelled '-1', the data are generated by

- $\boldsymbol{y}_{jO_1t_1} \sim \mathcal{N}(\boldsymbol{\mu}_t, \Sigma_0), \quad j = (N/2+1), ..., N, \ t_1 = 1, ..., T/2,$
  $\boldsymbol{y}_{jO_1t_2} \sim \mathcal{N}(\boldsymbol{\mu}_t, \Sigma_1), \quad j = (N/2+1), ..., N, \ t_2 = (T/2+1), ..., T,$
- $\boldsymbol{y}_{jO_2t_1} \sim \mathcal{N}(\boldsymbol{\mu}_t, \Sigma_0), \quad j = (N/2+1), ..., N, \ t_1 = 1, ..., T/2,$
  $\boldsymbol{y}_{jO_2t_2} \sim \mathcal{N}(\boldsymbol{\mu}_t, \Sigma_1), \quad j = (N/2+1), ..., N, \ t_2 = (T/2+1), ..., T,$
- $\boldsymbol{y}_{jO_3t_1} \sim \mathcal{N}(\boldsymbol{\mu}_t, \Sigma_1), \quad j = (N/2+1), ..., N, \ t_1 = 1, ..., T/2,$
  $\boldsymbol{y}_{jO_3t_2} \sim \mathcal{N}(\boldsymbol{\mu}_t, \Sigma_0), \quad j = (N/2+1), ..., N, \ t_2 = (T/2+1), ..., T,$
- $\boldsymbol{y}_{jO_4t_1} \sim \mathcal{N}(\boldsymbol{\mu}_t, \Sigma_1), \quad j = (N/2+1), ..., N, \ t_1 = 1, ..., T/2,$
  $\boldsymbol{y}_{jO_4t_2} \sim \mathcal{N}(\boldsymbol{\mu}_t, \Sigma_0), \quad j = (N/2+1), ..., N, \ t_2 = (T/2+1), ..., T.$

Under the settings of the simulation models, the first and third gene sets have changes in both the positive and negative groups, and the changes happen at time point $T/2$. For the second and fourth gene sets, the positive group has no change point and the negative group has changes at the $T/2$th time point. Therefore, the second and fourth gene sets are informative about the response label. We perform 50 simulations, and for each simulation we randomly select 70% subjects as the training set, and the remaining as the test set. We evaluate the performance of the proposed algorithm from two aspects: parameter inference and ROC curves. Firstly, Table S1 shows the results of parameter inference. In all cases, the value of b_2 is the largest of the four values corresponding to four parameters. The subject label is the result of different change points in gene sets 2 and 4. The values of b_2 and b_4 are consistent with the settings of the simulation models where for the gene set 2, the covariance matrix changes from Σ_0 to Σ_1, and for the gene set 4, the covariance matrix changes from Σ_1 to Σ_0. Secondly, the average ROC curves over 50 simulations of the classification results for each algorithm are shown in Figure S1. The results show that in all cases our algorithm outperforms the others. Especially when the sample size is small ($N \leq 50$), our algorithm performs better which benefits from the Bayesian model. When the sample size is large enough ($N \geq 100$ and $T \geq 80$), the advantage is not obvious. For the common used machine learning algorithms, it is also easy to distinguish the response labels in the case with enough samples.

Table S1. Estimations of parameters under different scenarios.

(N,T)	(20,40)	(20,80)	(20,150)	(50,40)	(50,80)	(50,150)	(100,40)	(100,80)	(100,150)
b_1	0.16<br>(0.03)	0.16<br>(0.02)	0.15<br>(0.01)	0.08<br>(0.01)	0.08<br>(0.01)	0.08<br>(0.01)	0.03<br>(0.04)	0.05<br>(0.03)	0.05<br>(0.02)
b_2	0.39<br>(0.06)	0.36<br>(0.04)	0.34<br>(0.03)	0.31<br>(0.05)	0.28<br>(0.04)	0.26<br>(0.02)	0.11<br>(0.10)	0.15<br>(0.10)	0.17<br>(0.05)
b_3	0.15<br>(0.02)	0.15<br>(0.02)	0.15<br>(0.01)	0.08<br>(0.02)	0.07<br>(0.01)	0.07<br>(0.01)	0.03<br>(0.04)	0.04<br>(0.03)	0.05<br>(0.02)
b_4	-0.13<br>(0.05)	-0.09<br>(0.03)	-0.07<br>(0.02)	-0.13<br>(0.04)	-0.11<br>(0.02)	-0.10<br>(0.01)	-0.07<br>(0.11)	-0.09<br>(0.08)	-0.10<br>(0.04)

Standard deviations are in the parenthesis.

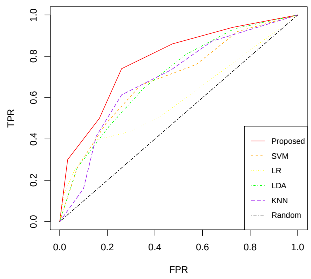

(a) $N = 20, T = 40$

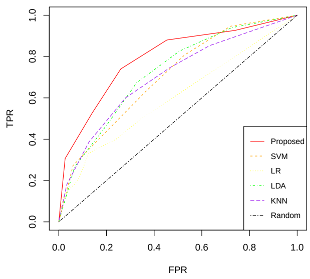

(b) $N = 20, T = 80$

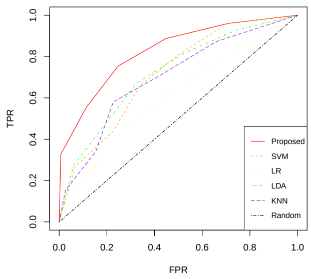

(c) $N = 20, T = 150$

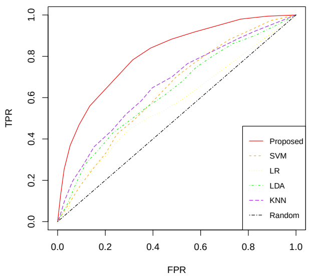

(d) $N = 50, T = 40$

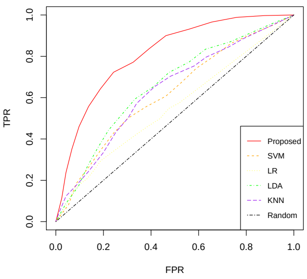

(e) $N = 50, T = 80$

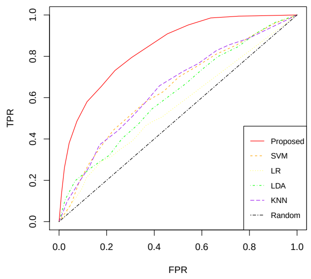

(f) $N = 50, T = 150$

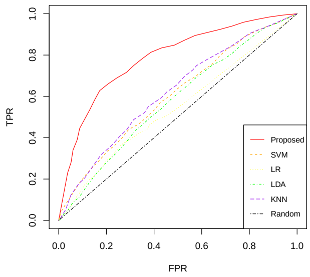

(g) $N = 100, T = 40$

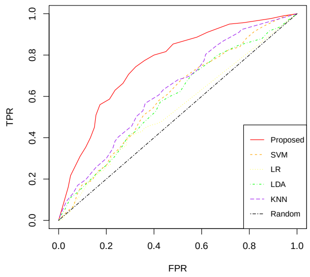

(h) $N = 100, T = 80$

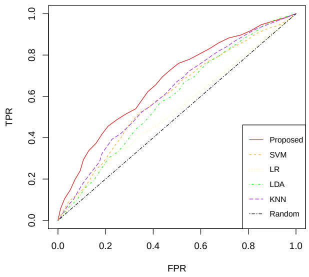

(i) $N = 100, T = 150$

Figure S1. ROC curves in different cases.

4. Analysis of the Effects of Gene Sets

In this section, we evaluated the performance of our algorithm based on different ways of grouping genes. We simulated three different datasets (dataset 1, dataset 2 and dataset 3) corresponding to three different cases to evaluate the robustness of our method under different cases. For each dataset, we randomly shuffled the features, and then grouped features using the defined criteria. We repeated this process six times for each dataset where each replication represented a way of grouping genes. Specifically,

- dataset 1: all genes in gene sets 1 and 2 were not related to the response, whereas all genes in gene sets 3 and 4 were informative about the response:
  - $\boldsymbol{x}_{iO_k t} \sim \mathcal{N}(\boldsymbol{\mu}_t, \Sigma_0), \quad t = 1, ..., T, \ k \in \{1, 2, 3, 4\},$

- $\boldsymbol{y}_{jO_l t} \sim \mathcal{N}(\boldsymbol{\mu}_t, \Sigma_0), \quad t = 1, ..., T, \ l \in \{1, 2\}$,
  $\boldsymbol{y}_{jO_m t_1} \sim \mathcal{N}(\boldsymbol{\mu}_t, \Sigma_0), \ t_1 = 1, ..., T/2, \ \boldsymbol{y}_{jO_m t_2} \sim \mathcal{N}(\boldsymbol{\mu}_t, \Sigma_1), \ t_2 = T/2 + 1, ..., T, \ m \in \{3, 4\}$,

- dataset 2: all genes in gene sets 1 and 2 were not related to the response, half of the genes in gene set 3 were related to the response, and all genes in gene set 4 were related to the response:
  - $\boldsymbol{x}_{iO_k t} \sim \mathcal{N}(\boldsymbol{\mu}_t, \Sigma_0), \quad t = 1, ..., T, \ k \in \{1, 2, 3, 4\}$,
  - $\boldsymbol{y}_{jO_l t} \sim \mathcal{N}(\boldsymbol{\mu}_t, \Sigma_0), \quad t = 1, ..., T, \ l \in \{1, 2\}$,
    $\boldsymbol{y}_{j\frac{O_3}{2}t} \sim \mathcal{N}(\boldsymbol{\mu}_t, \Sigma_0), \ t = 1, ..., T$,
    $\boldsymbol{y}_{j\frac{\bar{O}_3}{2}t_1} \sim \mathcal{N}(\boldsymbol{\mu}_t, \Sigma_0), \ t_1 = 1, ..., T/2, \boldsymbol{y}_{j\frac{\bar{O}_3}{2}t_2} \sim \mathcal{N}(\boldsymbol{\mu}_t, \Sigma_1), \ t_2 = T/2{+}1, ..., T$,
    $\boldsymbol{y}_{jO_4 t_1} \sim \mathcal{N}(\boldsymbol{\mu}_t, \Sigma_0), \ t_1 = 1, ..., T/2, \boldsymbol{y}_{jO_4 t_2} \sim \mathcal{N}(\boldsymbol{\mu}_t, \Sigma_1), \ t_2 = T/2{+}1, ..., T$,
- dataset 3: all genes in gene sets 1 and 2 were not related to the response, whereas half of the genes in gene set 3 and gene set 4 were related to the response:
  - $\boldsymbol{x}_{iO_k t} \sim \mathcal{N}(\boldsymbol{\mu}_t, \Sigma_0), \quad t = 1, ..., T, \ k \in \{1, 2, 3, 4\}$,
  - $\boldsymbol{y}_{jO_l t} \sim \mathcal{N}(\boldsymbol{\mu}_t, \Sigma_0), \quad t = 1, ..., T, \ l \in \{1, 2\}$,
    $\boldsymbol{y}_{j\frac{O_m}{2}t} \sim \mathcal{N}(\boldsymbol{\mu}_t, \Sigma_0), \ t = 1, ..., T, \ \boldsymbol{y}_{j\frac{\bar{O}_m}{2}t_1} \sim \mathcal{N}(\boldsymbol{\mu}_t, \Sigma_0), \ t_1 = 1, ..., T/2$,
    $\boldsymbol{y}_{j\frac{\bar{O}_m}{2}t_2} \sim \mathcal{N}(\boldsymbol{\mu}_t, \Sigma_1), \ t_2 = T/2 + 1, ..., T, \ m \in \{3, 4\}$.

$\frac{O_m}{2}$ represents half of the genes in gene set m, $\frac{\bar{O}_m}{2}$ is the complement set of $\frac{O_m}{2}$, Σ_0 is the identity matrix, $\Sigma_1 = \Sigma_0 + \rho \cdot \mathbb{1} - diag(\rho)$, $i \in \{1, ..., N/2\}$ and $j \in \{N/2{+}1, ..., N\}$. Here, the sample size $N = 100$, number of genes $G = 80$ and total number of time points $T = 40$. We partitioned these 80 genes into four gene sets indexed by O_1, O_2, O_3, and O_4, respectively, with each gene set containing 20 genes. To simplify the calculation, we picked $\rho = 0.7$ from $\{0.1, 0.3, 0.5, 0.7, 0.9\}$. For each replication, we shuffled the genes and then partitioned the genes into four groups according the simulation model. Under these simulation settings, the first and second gene sets had no information about the response. The third and fourth gene sets were related to the response. We summarized the results of the parameter estimations and prediction accuracy in Tables S2 and S3, respectively. Datasets $1-3$ correspond to cases $1-3$.

- Table S2 shows that the estimation of b depends on the groupings of genes. For cases 1 and 3, the values of b_3 and b_4 are larger than those of b_1 and b_2. As for case 2, where half of the genes in gene set 3 were related to the response and all the genes in gene set 4 were informative, it was difficult to identify gene set 3.

- Table S3 shows the results of AUC and prediction accuracy in different cases. We can see that different ways of grouping genes lead to different input information of our algorithm, which in turn, affect the prediction of the response. For case 3, 25%(20/80) of the genes were related to the response and they were split between two gene sets. Compared with cases 1 and 2, there was reduced prediction performance as expected.

These results are consistent with our expectations that the prior information as reflected in gene sets may impact the performance of our method where as a higher enrichment of signals in gene sets will lead to more accurate predictions.

Table S2. Estimations of parameter $\boldsymbol{b}$.

	$\boldsymbol{b}$	repeat 1	repeat 2	repeat 3	repeat 4	repeat 5	repeat 6
dataset 1 (case 1)	b_1	0.046	0.046	0.067	0.071	0.033	0.046
	b_2	0.070	0.037	0.010	0.052	0.034	0.055
	b_3	0.271	0.248	0.251	0.264	0.295	0.260
	b_4	0.244	0.273	0.275	0.241	0.220	0.265
dataset 2 (case 2)	b_1	0.024	0.043	0.045	0.061	0.064	0.098
	b_2	0.088	0.034	0.077	0.048	0.031	0.048
	b_3	-0.035	-0.073	-0.085	-0.089	-0.099	-0.119
	b_4	0.384	0.378	0.360	0.375	0.380	0.351
dataset 3 (case 3)	b_1	0.029	0.036	0.052	0.097	0.046	0.006
	b_2	0.048	0.038	-0.016	0.083	-0.010	-0.014
	b_3	0.323	0.392	0.329	0.267	0.276	0.259
	b_4	0.268	0.220	0.365	0.369	0.376	0.371

Table S3. AUC and prediction accuracy.

		repeat 1	repeat 2	repeat 3	repeat 4	repeat 5	repeat 6
dataset 1	AUC	0.978	0.964	0.996	0.956	0.969	0.973
	Accuracy	0.933	0.933	0.967	0.902	0.933	0.933
dataset 2	AUC	1.000	1.000	0.996	1.000	1.000	1.000
	Accuracy	1.000	1.000	0.966	1.000	1.000	1.000
dataset 3	AUC	0.653	0.622	0.609	0.738	0.658	0.693
	Accuracy	0.667	0.633	0.633	0.800	0.667	0.700

5. Challenge Results

Some challenge results related to this paper are shown in this section. In sub-challenge 2, the symptoms are measured as a binary outcome defined by whether an individual becomes symptomatic following exposure. Table S4 shows a part of prediction results within 0 or 24 hours. The p-values are computed via permutation test, and p-values have not been corrected for multiple testing. These seven teams are in the top seven.

Table S4. A part of challenge results.

	Hour 0		Hour 24	
Team	AUPR (p-value)	AUROC (p-value)	AUPR (p-value)	AUROC (p-value)
Schrodingers cat	0.949 (0.010)	0.838 (0.010)	0.953 (0.008)	0.863 (0.008)
cwruPatho	0.943 (0.016)	0.813 (0.020)	0.888 (0.102)	0.813 (0.019)
SSN DREAM Team	0.910 (0.055)	0.688 (0.118)	0.923 (0.036)	0.738 (0.061)
jdn	0.958 (0.008)	0.863 (0.008)	0.883 (0.115)	0.731 (0.067)
Chi Pak	0.675 (0.871)	0.275 (0.935)	0.858 (0.195)	0.663 (0.160)
USP team	0.844 (0.185)	0.674 (0.185)	0.844 (0.180)	0.674 (0.180)
Christofer Flinta	NA	NA	0.849 (0.214)	0.600 (0.266)

6. Figures

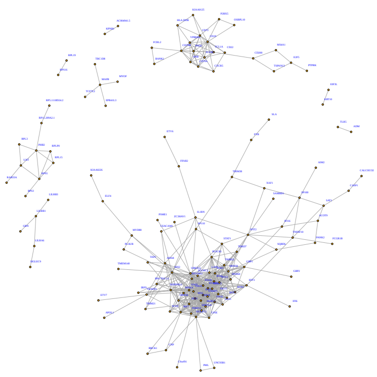

(a) Time 0

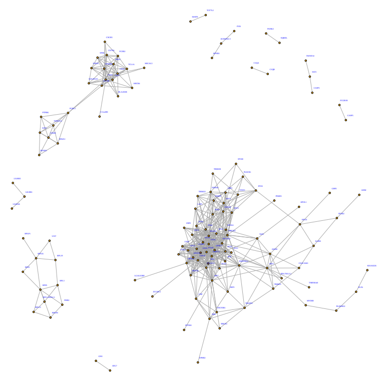

(b) Time 12

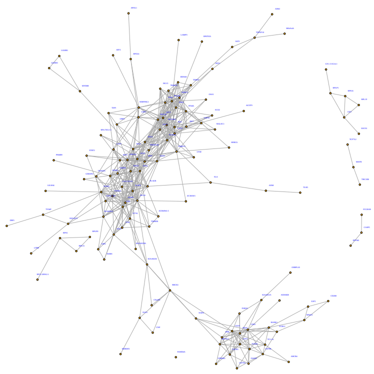

(c) Time 24

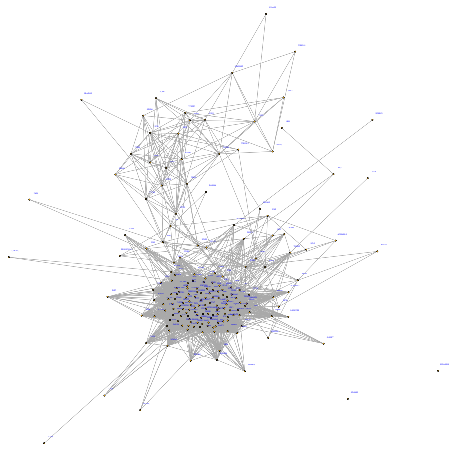

(d) Time 48

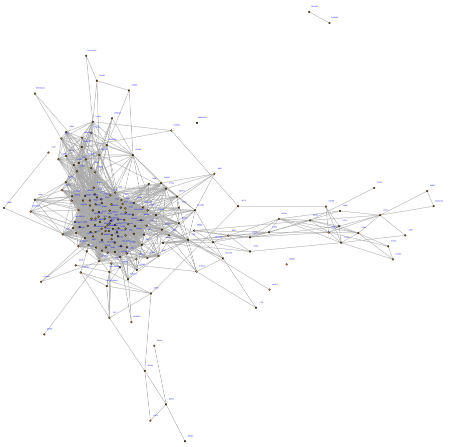

(e) Time 96

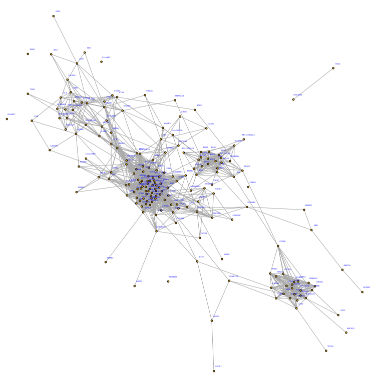

(f) Time 146

Figure S2. Co-expression networks of the 35th gene set at time points 0, 12, 24, 48, 96 and 146, respectively.

References

Bishop,C.M. (2007) *Pattern recognition and machine learning.* Springer, New York.

Chen,H. and Zhang,N. (2015) Graph-based change-point detection. *The Annals of Statistics*, **43**(1), 139-176.

Gönen,M. (2012) Bayesian efficient multiple kernel learning. *Proceedings of the 29th International Conference on Machine Learning*, Edinburgh, Scotland, The UK.
